# Size Effects of Highly Dispersed Bismuth Nanoparticles on Electrocatalytic Reduction of Carbon Dioxide to Formic Acid

**DOI:** 10.1021/jacs.3c04727

**Published:** 2023-06-15

**Authors:** Guangri Jia, Ying Wang, Mingzi Sun, Hao Zhang, Lejing Li, Yanbiao Shi, Lizhi Zhang, Xiaoqiang Cui, Tsz Woon Benedict Lo, Bolong Huang, Jimmy C. Yu

**Affiliations:** † Department of Chemistry, The Chinese University of Hong Kong, Shatin, New Territories, Hong Kong 999077, China; ‡ Department of Applied Biology and Chemical Technology, The Hong Kong Polytechnic University, Hung Hom, Kowloon, Hong Kong 999077, China; § State Key Laboratory of Automotive Simulation and Control, School of Materials Science and Engineering, Key Laboratory of Automobile Materials of MOE, Jilin University, Changchun 130012, China; ∥ School of Environmental Science and Engineering, Shanghai Jiao Tong University, Shanghai 200240, China

## Abstract

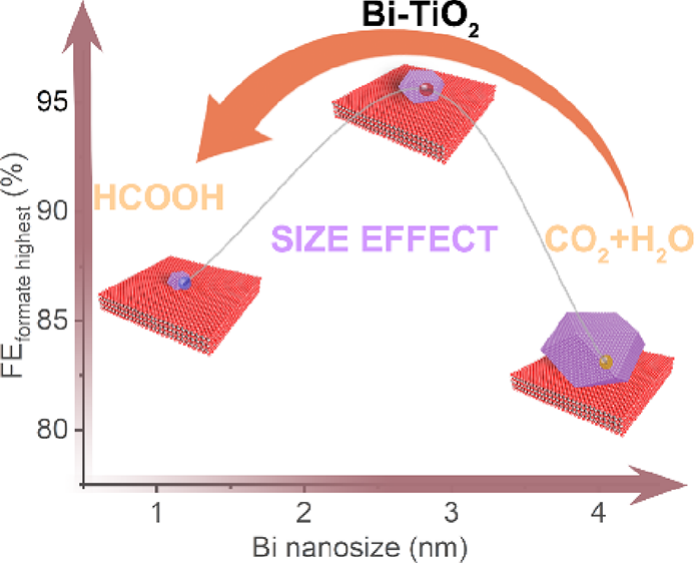

Electrocatalytic reduction of carbon dioxide into value-added chemical fuels is a promising way to achieve carbon neutrality. Bismuth-based materials have been considered as favorable electrocatalysts for converting carbon dioxide to formic acid. Moreover, size-dependent catalysis offers significant advantages in catalyzed heterogeneous chemical processes. However, the size effects of bismuth nanoparticles on formic acid production have not been fully explored. Here, we prepared Bi nanoparticles uniformly supported on porous TiO_2_ substrate electrocatalytic materials by in situ segregation of the Bi element from Bi_4_Ti_3_O_12_. The Bi-TiO_2_ electrocatalyst with Bi nanoparticles of 2.83 nm displays a Faradaic efficiency of greater than 90% over a wide potential range of 400 mV. Theoretical calculations have also demonstrated subtle electronic structural evolutions induced by the size variations of Bi nanoparticles, where the 2.83 nm Bi nanoparticles display the most active p-band and d-band centers to guarantee high electroactivity toward CO_2_RR.

## 1 Introduction

With the concept of carbon neutrality deeply rooted in the hearts of people, there is a growing desire to seek an effective way to reduce the concentration of CO_2_ in air to achieve the goal of carbon cycling.^1−3^ The electrocatalytic CO_2_ reduction reaction (CO_2_RR) is considered a promising approach to produce various value-added chemicals from CO_2_, water, and electrical energy obtained from wind, biomass, solar power, etc.^4−7^ Among the CO_2_RR products, formic acid has great market application prospects in chemical synthesis,^8,9^ fuel cells,^10,11^ and hydrogen storage carriers.^12−14^ However, how to efficiently produce formic acid by determining structure–property correlation remains challenging.^15,16^

Among various catalysts for electrocatalytic synthesis of formic acid, metal Bi-based materials are booming mainly due to their superior adsorption and activation of CO_2_, especially the effective conversion of intermediate OCHO* to HCOO^–^ and the inhibition of competing products of CO and H_2_.^17−20^ Recently, the typical results have been shown in a number of examples. For instance, structural engineering of Bi metal at the nanoscale shows a high Faradaic efficiency (FE) due to the enlarged surface area and abundant unsaturated Bi sites for CO_2_ adsorption.^21,22^ The alloyed Bi metal can effectively improve the FE of formic acid by adjusting the electron states to increase the adsorption and activation of intermediates.^23−26^ In addition, due to the adjustable electronic and catalytic properties, the size effect has been widely applied in heterogeneous catalysis.^27−29^ Especially for CO_2_RR, the selection of the reaction pathway and the activity of products can be greatly affected by modulating the adsorption energy between the active sites and intermediates through size effects.^30−32^ However, few explorations have been performed on the Bi size dependence for CO_2_ reduction due to intrinsically different catalytic performances with different sizes. The crucial challenges include (1) homogeneity of Bi metal sites with different sizes, (2) exploration of the interaction between metals and substrates, and (3) accumulation of the low melting point of Bi metals in the process of binding with the substrate. Therefore, it is of great significance to explore the size effect and understand its structure–activity relationship for the rapid production of formate by selective CO_2_ reduction.

Here, we present active Bi-TiO_2_ with uniform Bi nanoparticles supported on porous TiO_2_ via in situ Bi segregation from Bi_4_Ti_3_O_12_. The size of Bi nanoparticles could be engineered by regulating the segregation rate of Bi according to the annealing temperature. The optimal Bi-TiO_2_ shows the highest FE of formate (FE_formate_) up to 95.6% at −1.0 V (vs RHE) by suppressing H_2_ and CO formation in a 0.1 M KHCO_3_ electrolyte using an H-type cell. The high FE_formate_ (>90%) is maintained at a wide potential window of 400 mV. These are attributed to the abundantly exposed active Bi sites with suitable adsorption energy for CO_2_RR intermediates and efficient interactions between Bi and TiO_2_. Density functional theory (DFT) calculations have indicated that the size of Bi nanoparticles is significant to modulate the electroactivity and hence the CO_2_RR performances. Bi-TiO_2_-700 with medium sizes of Bi nanoparticles exhibits the highest electroactivity due to upshifting p-band and d-band centers. The abundant interfaces in Bi-TiO_2_ improve the electroactivity and ensure the strong adsorption of CO_2_ to lower the energy costs of CO_2_RR.

## 2 Results and Discussion

### 2.1 Synthesis and Characterization of Bi-TiO_2_

**Figure 1:**
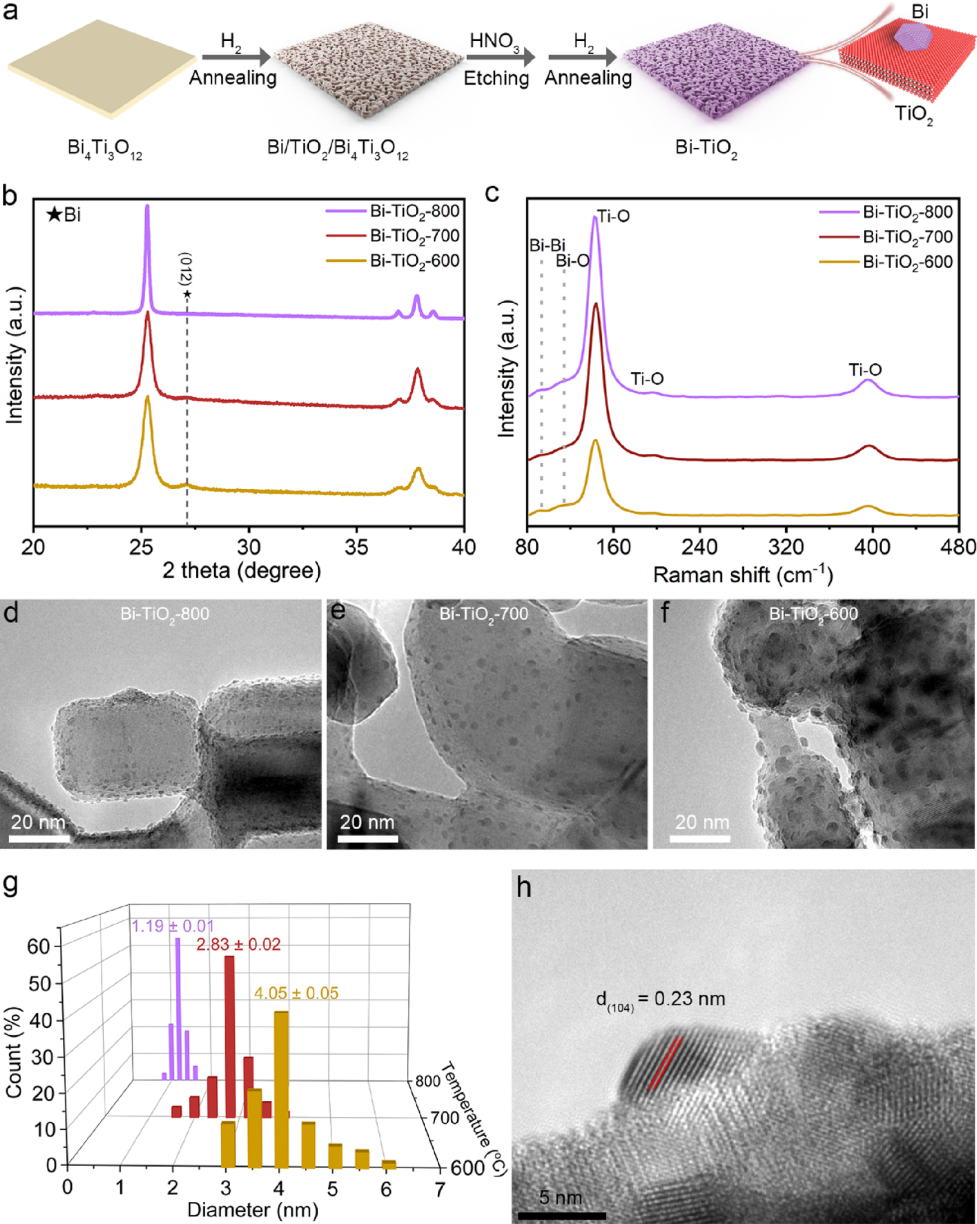
(a) Synthesis scheme of the electrocatalyst. (b) XRD patterns and (c) Raman spectra of Bi-TiO_2_-600, Bi-TiO_2_-700, and Bi-TiO_2_-800. (d–f) TEM images of Bi-TiO_2_-800, Bi-TiO_2_-700, and Bi-TiO_2_-600. (g) Statistical size distribution of nanoparticles of Bi of Bi-TiO_2_-600, Bi-TiO_2_-700, and Bi-TiO_2_-800. (h) HRTEM image of Bi-TiO_2_-700.

Based on the idea of in situ segregation, a schematic diagram illustrates the synthesis of Bi-TiO_2_ with different sizes of Bi (Figure 1a). First, two-dimensional Bi_4_Ti_3_O_12_ nanosheets were prepared by solid-phase synthesis. Part of Bi is removed from Bi_4_Ti_3_O_12_ after the initial annealing treatment, which is mainly to avoid the uneven Bi nanoparticles in the final synthesized Bi-TiO_2_. Then, the Bi segregated by the initial annealing is etched with an acid. Finally, Bi-TiO_2_ is obtained by annealing the acid-etched sample in a hydrogen atmosphere. We found after temperature optimization that the precursor could not be completely reduced at low temperatures of 300–400 °C. Moreover, at a high temperature of 900 °C, the porous structure of the nanosheet would be destroyed, leading to smaller Bi nanoparticles. Products obtained under these conditions are not as effective for electrocatalytic CO_2_ reduction. The X-ray diffraction (XRD) pattern shows that the crystal structure of Bi-TiO_2_ at different temperatures is mainly anatase phase TiO_2_ and Bi metal (Figure 1b and Figure S1). The difference is that the full width at half-maxima of Bi gradually increases and the relative strength gradually decreases with the increase of temperature. The results are attributed to that abundant nucleation sites are quickly formed on the surface of TiO_2_, resulting in a gradual decrease in the size of Bi nanoparticles with an increase in temperature. Moreover, Raman spectroscopic results show that there is a Bi–O structure (112.3 cm^–1^) in addition to the Bi–Bi (91.1 cm^–1^) structure, which is mainly due to the easy oxidation of the Bi surface (Figure 1c). Based on the premise of segregation of the Bi element from the two-dimensional precursor Bi_4_Ti_3_O_12_, there are many pore structures on the surface of the eventually formed Bi-TiO_2_, which can be proven by scanning electron microscope (SEM) images and transmission electron microscope (TEM) images (Figures S2–S5). Also, it is found that the size of the holes increases with respect to the annealing temperature accordingly. After magnifying the local morphologies, the TEM images show that the Bi nanoparticle sizes are concentrated in 1.15 (Bi-TiO_2_-800), 2.83 (Bi-TiO_2_-700), and 4.05 nm (Bi-TiO_2_-600) due to different nucleation rates (Figure 1d–g). A high-resolution TEM (HRTEM) image confirms that Bi nanostructures are tightly bound to TiO_2_ (Figure 1h). Energy-dispersive X-ray spectroscopy (EDS) depicts that the atomic percent (at. %) of Bi is 2.89, 2.84, and 2.86 at. % for Bi-TiO_2_-600, Bi-TiO_2_-700, and Bi-TiO_2_-800, respectively (Table S1). Elemental mapping further shows that Bi is finely distributed around TiO_2_ (Figure S6).

**Figure 2:**
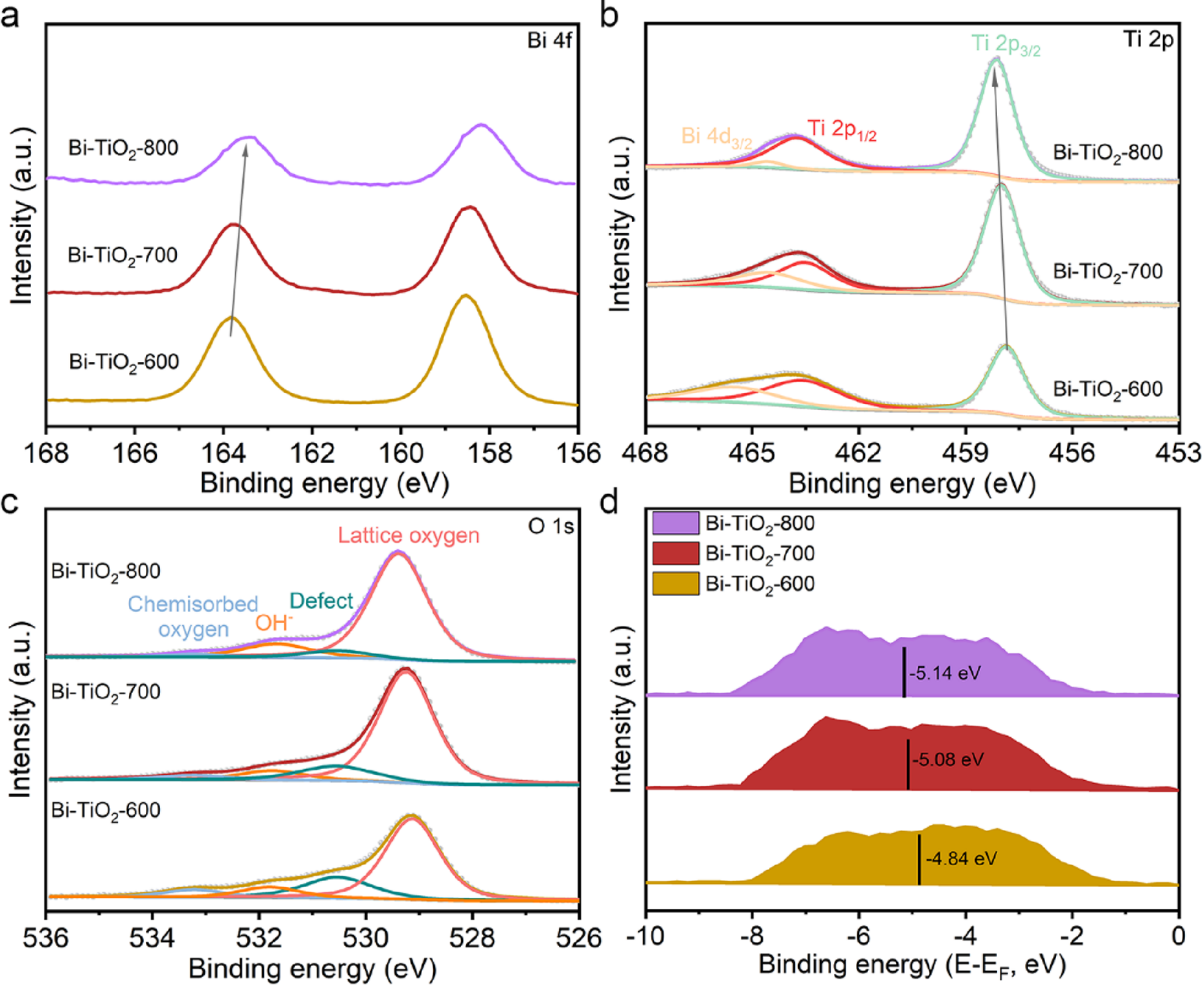
XPS spectra of (a) Bi 4f, (b) Ti 2p, and (c) O 1s. (d) d-Band center extrapolated from VB-XPS of Bi-TiO_2_-600, Bi-TiO_2_-700, and Bi-TiO_2_-800.

X-ray photoelectron spectroscopy (XPS) was used to investigate the chemical states of Ti, O, and Bi of the catalyst surface (Figure S7). We first investigated the Bi 4f spectrum (Figure 2a and Figure S8). When the annealing temperature is 600 °C, the XPS peak pair at 158.4 and 163.9 eV corresponds to the Bi^3+^ species, which is mainly due to the oxidation of the Bi surface by air combined with XRD and Raman data.^33,34^ With the temperature increasing, the Bi 4f peaks shift to low binding energy due to the Bi element gradually reduced and the interaction between Bi and TiO_2_. Subsequently, we studied Ti 2p XPS, and the valence states of Ti 2p gradually shift to high binding energy with temperature increasing, which is attributed to electron transfer from TiO_2_ to Bi atoms in the interface (Figure 2b and Figure S9). Furthermore, the O 1s peaks also show the presence of possible oxygen vacancy defects in Figure 2c,^35^ which has a positive effect on the adsorption of molecules. The intensity of oxygen vacancy gradually reduces with increasing annealing temperature, attributing to the diffusion effect at high temperatures and the strong interaction between Bi and TiO_2_, which impeded further reduction of TiO_2_ (Figures S10 and S11). We also obtained the d-band center of Bi-TiO_2_-600, Bi-TiO_2_-700, and Bi-TiO_2_-800 through VB-XPS (Figure 2d), which can be used as a “descriptor” to describe the adsorption energy of an adsorbed molecule on transition metal sites. Surprisingly, the d-band center gradually downshifts with the decrease of Bi particle sizes of Bi-TiO_2_-600, Bi-TiO_2_-700, and Bi-TiO_2_-800, which is due to compressive strain governed by the Young–Laplace equation.^36^ Compared with Bi-TiO_2_-600, the d-band center of Bi-TiO_2_-700 downshifts, which decreases the binding strength between the intermediates and the Bi metal sites, which could improve the antipoisoning capability of the electrocatalysts by donating more electrons to the Bi–CO antibonding orbital. On the contrary, the d-band center of Bi-TiO_2_-700 upshifts compared with that of Bi-TiO_2_-800, which would enhance the adsorption of OCHO* oxygen-containing intermediates. Therefore, these results suggest that a reasonable d-band center is needed for catalytic CO_2_ reduction.

### 2.2 CO_2_RR Performance of Bi-TiO_2_

**Figure 3:**
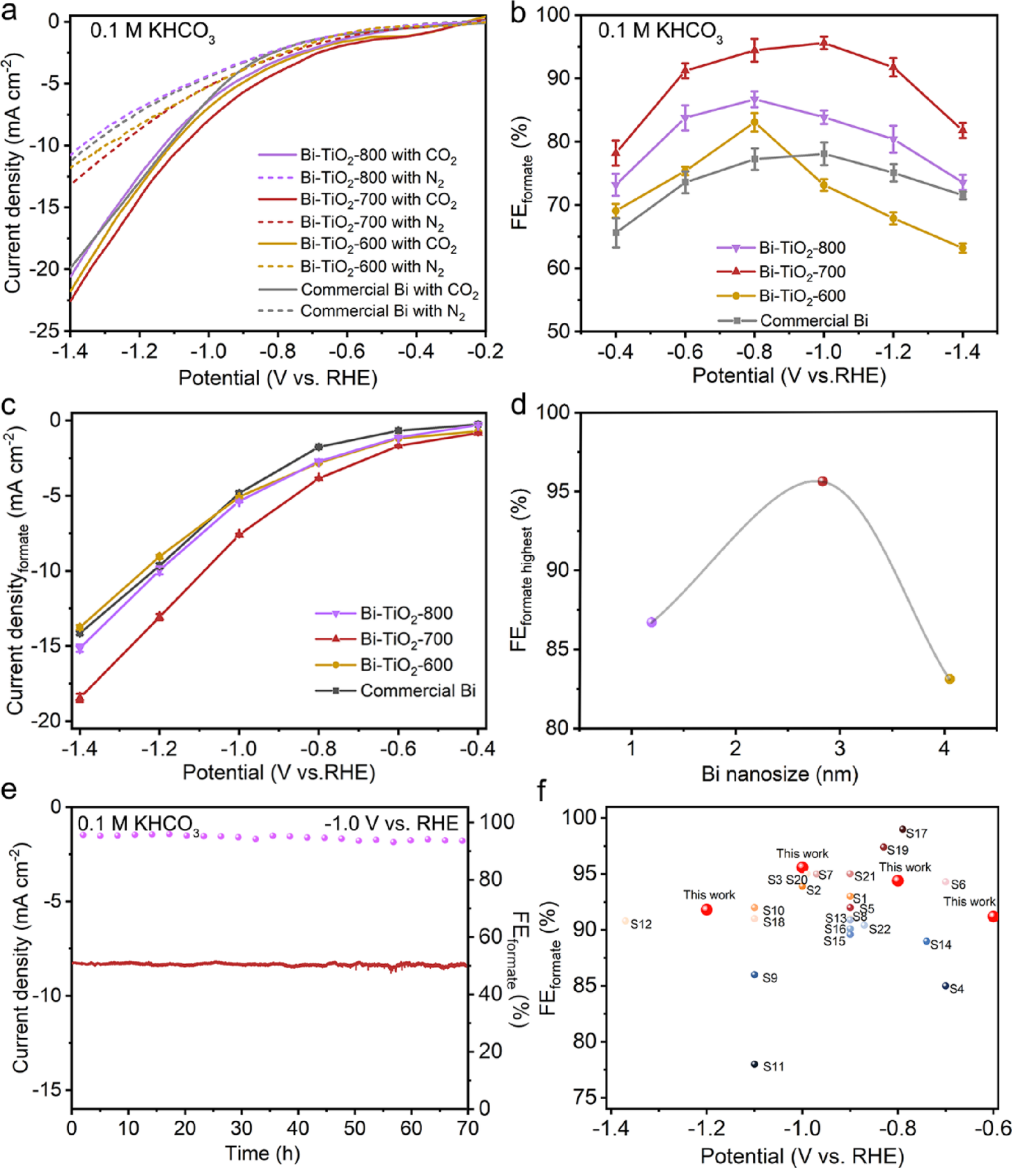
(a) LSVs of Bi-TiO_2_-600, Bi-TiO_2_-700, and Bi-TiO_2_-800 in CO_2_-saturated and Ar-bubbled 0.1 M KHCO_3_ in an H-type cell. (b) Comparison of FEs of formate of the prepared Bi-TiO_2_-600, Bi-TiO_2_-700, and Bi-TiO_2_-800 in an H-type cell at various applied potentials. (c) Formate partial current density and (d) dependence of activity with Bi nanosize. (e) Durability test of Bi-TiO_2_-700 in 0.1 M KHCO_3_ in an H-type cell configuration for 70 h. (f) Comparison of FEs at different applied potentials with previously reported electrocatalysts (see Table S2 for details of the reported data).

First, the catalytic performance of Bi-TiO_2_ electrocatalysts for CO_2_RR was evaluated in a gastight two-compartment H-cell with CO_2_-saturated 0.1 M KHCO_3_ as the electrolyte. Compared with linear sweep voltammetry (LSV) in the Ar-purged catholyte, the current densities measured in the CO_2_-saturated catholyte show a dramatic increase, indicating that CO_2_ is more favorably reduced than the HER in the CO_2_-purged catholyte (Figure 3a). Moreover, the resultant products were quantitatively analyzed via online gas chromatography (GC) for gas and ^1^H nuclear magnetic resonance (^1^H NMR) spectroscopy for liquid (Figure S12). Bi-TiO_2_-700 material indicates the highest Faradaic efficiency of formate (FE_formate_) of 95.6 ± 1.0% at −1.0 V (vs RHE) (Figure 3b and Figure S13), which is better than that of commercial Bi metal (maximum FE_formate_ of 78.0 ± 1.8% at −1.0 V vs RHE) and most Bi-based materials (Figure 3f and Tables S2 and S3). The FE_formate_ is more than 90% over a wide potential range of 400 mV (−0.6 to −1.0 V vs RHE) for Bi-TiO_2_-700, which might be attributed to the efficient mass transport of the two-dimensional porous structure, the densely rich active sites, and the moderate electron state to balance the adsorption of two competing *OCHO and COOH*. Moreover, the highest partial current density of formate is achieved by Bi-TiO_2_-700 over a wide potential range (−0.4 to −1.4 V vs RHE) (Figure 3c). For comparison, the products of Bi-TiO_2_-600 and Bi-TiO_2_-800 were also quantitatively analyzed and evaluated to further manifest the synergistic effects of Bi nanoparticles and the TiO_2_ substrate. When a larger Bi nanoparticle size of Bi-TiO_2_–600 is obtained and applied to CO_2_RR, the FE_formate_ is lower than that of Bi-TiO_2_-700, which may be due to the lower adsorption energy for the *OCHO intermediate. However, for Bi-TiO_2_-800, a smaller Bi nanosize also leads to weaker activity of formate than that of Bi-TiO_2_-700, which is due to the poisoning of CO; the smaller electrochemical active surface area, larger electrochemical impedance, and hydrophilicity limited the adsorption of CO_2_ (Figures S14–S16). In addition, the flow cell test was carried out to achieve a larger current density in a 1 M KOH electrolyte using a gas diffusion electrode (Figure S17). The partial current density of formate is greatly improved up to 102.6 mA cm^–2^ at −1.0 V with the highest FE_formate_ of 95.8%. Moreover, the FE_formate_ is also maintained at a high level above 90% over a wide potential range (−0.6 to −1.2 V). A long-term performance is carried out on Bi-TiO_2_-700. The stability testing was evaluated at a constant potential of −1.0 V. The FE_formate_ maintains above 95%, and the current density presents a stable value of 8.4 ± 0.2 mA cm^–2^ for 70 h with negligible degradation (Figure 3e). Microscopy characterization and the elemental Bi content (2.79 at. %) of Bi-TiO_2_-700 after long-term electrolysis indicate the high stability of the samples (Figure S18).

### 2.3 In Situ Raman and ATR-FTIR Analysis

**Figure 4:**
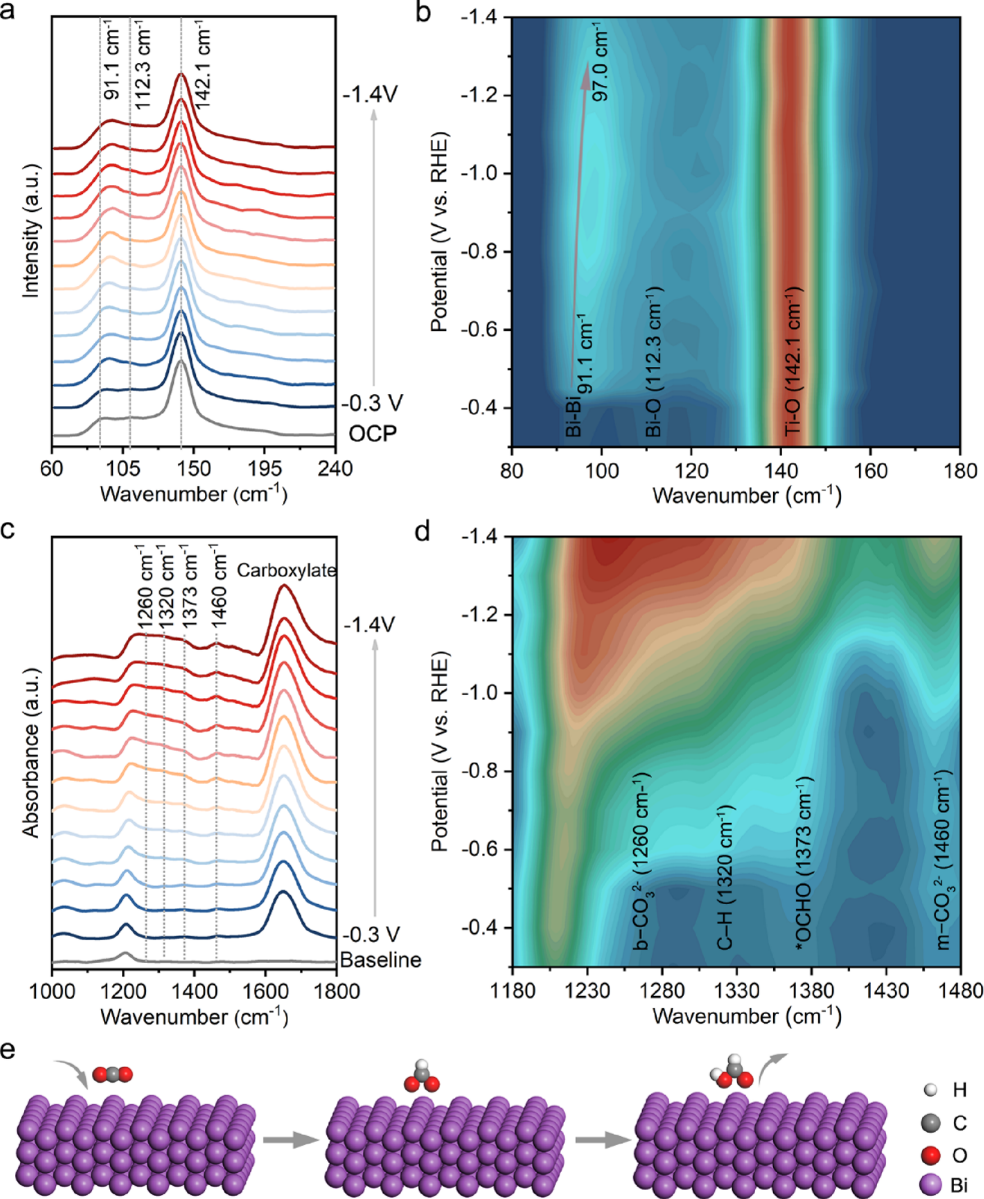
(a,b) In situ Raman spectra and (c,d) in situ ATR-FTIR spectra collected of Bi-TiO_2_-700 at different applied potentials from −0.3 to −1.4 V vs RHE in a 0.1 M KHCO_3_ electrolyte. (e) Possible pathways of the *OCHO pathway to produce formate in electrochemical CO_2_ reduction. H, C, O, and Bi atoms are represented by white, gray, red, and purple spheres, respectively.

In situ Raman analysis is a stronger tool to investigate the catalyst surface structure during the CO_2_ reduction process. At an open circuit potential (OCP), two typical Raman peaks at 91.1 and 112.3 cm^–1^ are attributed to the Bi–Bi stretching vibration of metal Bi and the Bi–O stretching vibration of BiO_*x*_ from surface oxidation of the catalyst in Figure 4a,b.^37,38^ A strong Raman signal at 142.1 cm^–1^ belongs to Ti–O and is unchanged throughout the reduction process.^38,39^ The band at 112.3 cm^–1^ disappears gradually, and the vibration signal of metallic Bi (91.1 cm^–1^) shows an increased trend at −0.4 V, indicating that the catalyst begins to reduce to metallic Bi. With gradually increasing cathodic potential to −1.4 V versus RHE, the peak of Bi–Bi then shifts from 91.1 to 97.0 cm^–1^ at more negative potentials, which presents that the Bi–Bi structure is bonded with the intermediate (*OCHO).^40^

To further unravel the catalytic intermediates and the reaction pathways, in situ attenuated total reflectance-Fourier transform infrared (ATR-FTIR) measurement was conducted to study the electrocatalyst Bi-TiO_2_-700 with excellent electrocatalytic performances for CO_2_RR at applied potentials from −0.3 to 1.4 V vs RHE. As indicated in Figure 4c,d, a characteristic signal located at 1373 cm^–1^ appears and gradually increases with applied potentials varied from −0.4 to −1.4 V.^25,41,42^ This band is ascribed to the vibration of O–C–O of *OCHO species, which is an important intermediate of formate (Figure 4e). The peak located at 1320 cm^–1^ is ascribed to the C–H deformation vibration in adsorbed OCHO* species.^43^ The peaks at 1268 and 1460 cm^–1^ are from bidentate carbonate (b-CO_3_^2–^) of the asymmetric OCO stretches and monodentate carbonate (m-CO_3_^2–^) groups.^44−46^ The band at 1648 cm^–1^ corresponding to carboxylate increases with applied potentials, which mainly originates from the surface adsorption of the electrode at high potentials from the KHCO_3_ electrolyte. Moreover, no typical peaks of CO* (1900 to 2100 cm^–1^) are detected during the electrocatalytic process, demonstrating the negligible generation of CO on the surface of the electrocatalyst (Figure S19).^47,48^

### 2.4 Theoretical Calculation Analysis

**Figure 5:**
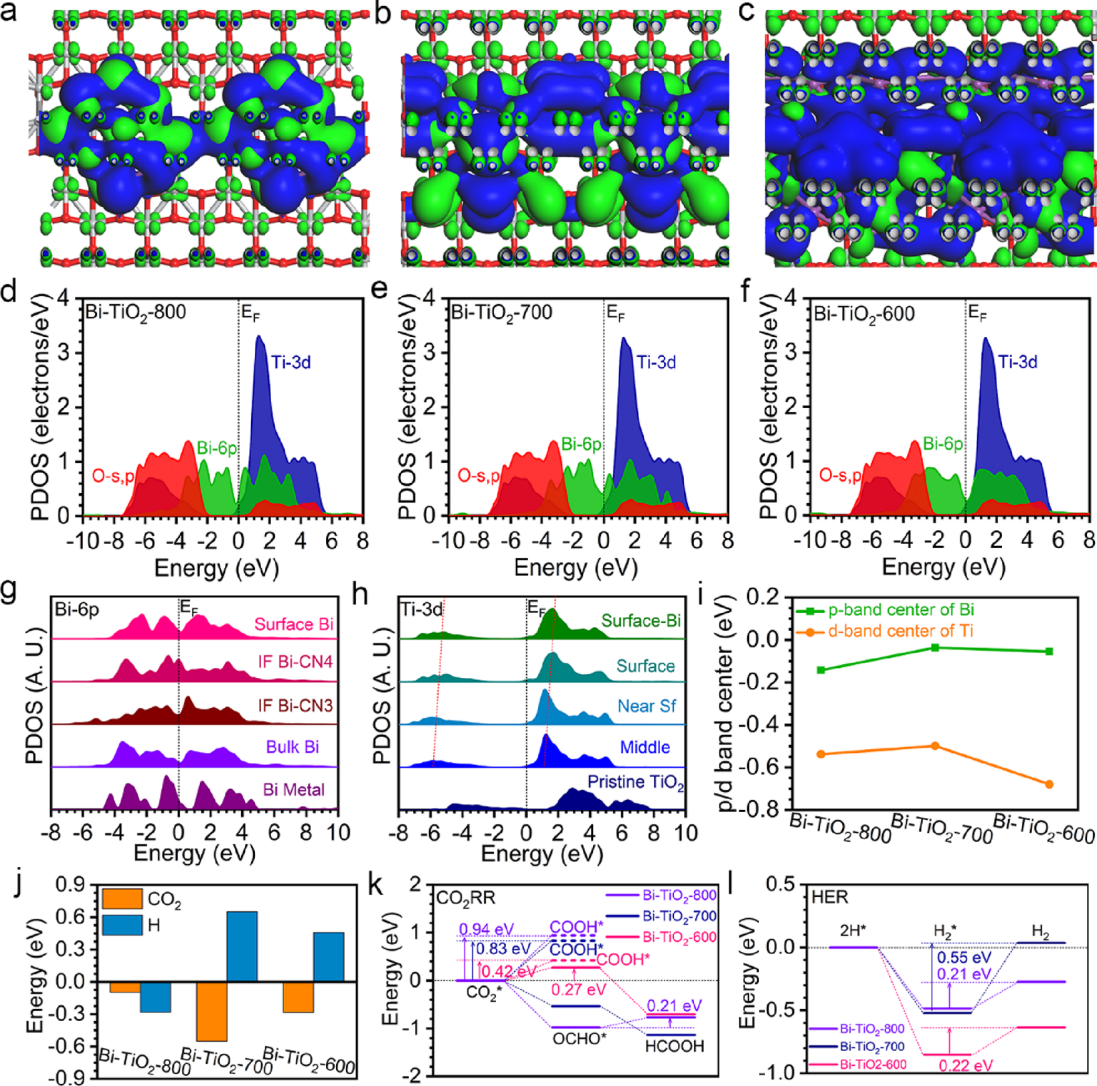
3D contour plot of electronic distributions near the Fermi level on (a) Bi-TiO_2_-800, (b) Bi-TiO_2_-700, and (c) Bi-TiO_2_-600. Purple balls = Bi, gray balls = Ti, and red balls = O. Blue isosurface = bonding orbitals, and green isosurface = antibonding orbitals. Bi nanoparticles have 10, 14, and 26 atoms for Bi-TiO_2_-800, Bi-TiO_2_-700, and Bi-TiO_2_-600, respectively. PDOS of (d) Bi-TiO_2_-800, (e) Bi-TiO_2_-700, and (f) Bi-TiO_2_-600. Site-dependent PDOS of (g) Bi 6p and (h) Ti 3d in Bi-TiO_2_-700. (i) p-Band and d-band center evolutions. (j) Adsorption energy comparisons of CO_2_* and H*. (k) Reaction energy change of CO_2_RR on Bi-TiO_2_. (l) Reaction energy change of H_2_ generation on Bi-TiO_2_.

To further understand the electroactivity evolution induced by the size of Bi nanoparticles, we have carried out theoretical calculations based on DFT. Three sizes of Bi nanoparticles including 10, 14, and 26 atoms have been considered on TiO_2_ as Bi-TiO_2_-800, Bi-TiO_2_-700, and Bi-TiO_2_-600, respectively. The sizes of Bi nanoparticles are 0.59, 0.80, and 1.19 nm, respectively, based on the computational capability of DFT calculations. From the electronic distributions near the Fermi level (*E*_F_), we notice that Bi strongly dominates the bonding and antibonding orbitals to contribute to the electroactivity (Figure 5a–c). Ti sites display strong contributions to antibonding orbitals, and O sites show limited contributions to the bonding orbitals near the interface with Bi nanoparticles. For all the Bi nanoparticles, the bonding orbitals are dominated by the low coordinated surface sites. As the size of the Bi nanoparticles increases, the surface electron-rich feature becomes more evident, which promotes electron transfer. To further understand the detailed contributions of the electronic structure, we further demonstrate the projected partial density of state (PDOSs) (Figure 5d–f). The overall electronic structures are similar for Bi-TiO_2_ with different sizes of Bi nanoparticles. Notably, Bi 6p orbitals have shown the main contributions near the *E*_F_, which varies with the Bi nanoparticle sizes. As the size increases, we notice that Bi 6p orbitals display the highest electron density near *E*_F_ for the medium Bi nanoparticles of Bi-TiO_2_. This indicates that the electron transfer capability is subtly affected by the nanoparticle sizes. The O s and p and Ti 3d orbitals locate at the lowest position with good overlapping, playing as the electron reservoir during the CO_2_RR. The electron transfer is facilitated by the strong interfacial orbital coupling between TiO_2_ and Bi nanoparticles. The site-dependent Bi 6p orbitals are further demonstrated to reveal the electronic structure evolutions (Figure 5g). Notably, compared to the bulk Bi metal, the electronic structures of Bi nanoparticles are evidently changed, where Bi 6p orbitals become broadened and cross the *E*_F_, supporting an improved electroactivity. From the bulk site inside Bi nanoparticles to the surface low coordinated Bi sites, it is noted that the electron density near *E*_F_ gradually increased. In particular, the interfacial Bi sites with more bonding exhibit the highest electron density near *E*_F_, which guarantees the efficient interfacial electron transfer between TiO_2_ and Bi nanoparticles. On the other side, the electronic structures of the TiO_2_ surface are also modulated due to the interactions with the Bi nanoparticles (Figure 5h). Compared to bulk anatase TiO_2_, the surface has displayed significantly downshifted 3d orbitals. From the bulk to the surface, Ti 3d orbitals deliver an upshifting trend, indicating the increasing d-band center. For the surface Ti at the interfacial region, Ti 3d orbitals are strongly modulated. Therefore, this indicates that the formation of the interface in Bi-TiO_2_ leads to electronic structure modulations, especially for the Bi nanoparticles. Then, we compared the d-band and p-band centers of Bi-TiO_2_-600, Bi-TiO_2_-700, and Bi-TiO_2_-800 (Figure 5i). As the size of the Bi nanoparticle increases, the d-band center upshifts in Bi-TiO_2_-700, which is consistent with the experimental characterization results. The further increases of Bi nanoparticle size result in the decrease of surface unsaturated Ti sites, which leads to the downshift of the d-band center. The medium-size Bi-TiO_2_-700 exhibits an optimum electron transfer efficiency for surface Bi sites and thus shows the highest electroactivity for CO_2_RR. These findings clearly correlate the electroactivity of Bi-TiO_2_ with the size of Bi nanoparticles.

In order to unravel the electrocatalysis process, the adsorption energies of key reactants are compared (Figure 5j). Apparently, although all Bi-TiO_2_ catalysts show preferred CO_2_ adsorption, it is most favored on Bi-TiO_2_-700 with the lowest energy to support the efficient CO_2_RR. Meanwhile, proton adsorption is not favored on the Bi-TiO_2_-700 and Bi-TiO_2_-600, lowering the efficiency of the competitive HER process. However, Bi-TiO_2_-800 delivers stronger proton adsorption than CO_2_, which potentially suppresses the CO_2_RR performance by the HER process. It is worth noting that the adsorption of CO_2_ for all the Bi-TiO_2_ is mostly preferred on the interfacial sites, which confirms that the increased interfacial regions largely promote the CO_2_RR. To understand the reaction process, we compared the CO_2_RR reduction energy changes (Figure 5k). Notably, Bi-TiO_2_-700 shows a continuous downshifting reaction trend for the HCOOH formation, indicating superior selectivity and efficiency of CO_2_RR. In comparison, Bi-TiO_2_-800 and Bi-TiO_2_-600 deliver evident energy barriers of 0.21 and 0.27 eV for OCHO* and HCOOH formation steps, respectively. As the competitive reaction pathway of HCOOH, the reaction energy costs of COOH* are also supplied, which is the key intermediate for the CO pathway. For all the Bi-TiO_2_ catalysts, the generation of COOH* requires much higher energy costs than the OCHO*. This leads to high selectivity toward the formation of HCOOH. In addition to the proton binding energies, the further hydrogen generation process is also studied (Figure 5l). The conversion from 2H* to H_2_* is spontaneous, while the desorption of the formed H_2_ meets a large energy barrier for all Bi-TiO_2_ nanocatalysts. Bi-TiO_2_-700 has an energy barrier of 0.51 eV with a positive reaction trend, which guarantees low H_2_ generation during CO_2_RR. The energy barriers for Bi-TiO_2_-600 and Bi-TiO_2_-800 of hydrogen generation are similar to CO_2_RR, leading to the potential competition between H_2_ and HCOOH generation. These results indicate that the medium Bi nanoparticles on TiO_2_ supply the optimal electronic structures, which is the key to achieve high selectivity of the HCOOH during CO_2_RR.

## 3 Conclusions

We have successfully developed a novel uniform nanoparticle catalyst strategy that relies on the distribution of target sizes of Bi on a stable TiO_2_ porous substrate as a highly active electrocatalyst for CO_2_ reduction. In situ segregation of Bi on TiO_2_ ensures the stability of the catalyst during the reaction. Depending on the nucleation rate at different temperatures, the sizes of Bi nanoparticles are different, which determines the different surface energies, and thus have different adsorption energy barriers for reaction intermediates. The resultant 2.83 nm Bi has the optimal adsorption and activation. DFT calculations have unraveled that the electronic structure evolutions of Bi-TiO_2_ experience a volcano trend based on the size of Bi nanoparticles, where Bi-TiO_2_-700 with medium Bi nanoparticles achieves the highest electroactivity. The optimized electroactivity in Bi-TiO_2_-700 not only shows the strongest CO_2_ adsorptions but also successfully suppresses the CO and H_2_ generation to realize the superior CO_2_RR with high selectivity to HCOOH.

## Supporting Information Available

Experimental details; DFT calculation details; morphology and structure characterizations; electrochemical data (PDF)
