# Supplementary material for: Size Effects of Highly Dispersed Bismuth Nanoparticles on Electrocatalytic Reduction of Carbon Dioxide to Formic Acid
Source: J Am Chem Soc. 2023 Jun 15;145(25):14133–42. doi: 10.1021/jacs.3c04727 (PMC10311520; doi:10.1021/jacs.3c04727)
Supplement: Supplementary file 1 — ja3c04727_si_001.pdf [file ja3c04727_si_001.pdf]

Supporting Information

Size Effects of Highly Dispersed Bismuth Nanoparticles on Electrocatalytic Reduction of Carbon Dioxide to Formic Acid

Guangri Jia[a#], Ying Wang[c#], Mingzi Sun[b#], Hao Zhang[b], Lejing Li[a], Yanbiao Shi[d], Lizhi Zhang[d], Xiaoqiang Cui[c], Tsz Woon Benedict Lo[b*], Bolong Huang[b*], and Jimmy C. Yu[a*]

[a] Department of Chemistry, The Chinese University of Hong Kong, Shatin, New Territories, Hong Kong 999077, China

[b] Department of Applied Biology and Chemical Technology, The Hong Kong Polytechnic University, Hung Hom, Kowloon, Hong Kong 999077, China

[c] State Key Laboratory of Automotive Simulation and Control, School of Materials Science and Engineering, Key Laboratory of Automobile Materials of MOE, Jilin University, Changchun 130012, China

[d] School of Environmental Science and Engineering, Shanghai Jiao Tong University, Shanghai 200240, China

[#] These authors contributed equally to this paper.

[*] Email: jimyu@cuhk.edu.hk (J. C. Yu), bhuang@polyu.edu.hk (B. Huang), benedict.tw.lo@polyu.edu.hk (T. W. B. Lo)

Methods

1. Chemicals and materials

Commercial P25 was purchased from the Sinopharm Chemical Reagent Co., Ltd (China). Bi_2O_3 was obtained from Aladdin (USA). HNO_3 was purchased from VWR (France). $KHCO_3$ was purchased from Sigma-Aldrich. Deionized water (DI) was used throughout the experiment. All the chemicals were used without further purification.

2. Synthesis of catalyst

For synthesis of $Bi_4Ti_3O_{12}$ through a traditional solid-state reaction method, 0.5825 g of Bi_2O_3 and 0.15 g of P25 (mol ratio is 2:3) were added in a mixture of 7.455 g of KCl and 5.844 g of NaCl. The mixture was ground for 15 minutes in a mortar and annealed at 800 °C and 5 °C min^{-1} for 12 h in air. The sample was then collected and washed with DI water and ethanol for three times, and then dried at 60 °C for 8 h. For the preparation of Bi-TiO_2, firstly, the prepared $Bi_4Ti_3O_{12}$ was annealed at 500 °C for 0.5 h with a rate of 15 °C min^{-1} in H_2 atmosphere (5% H_2 and 95% Ar). Then, the sample was etched to remove Bi metal in 5 M HNO_3 for 0.5 h. Then, we found that different synthesis temperatures lead to different sizes of Bi nanoparticle electrocatalysts due to different nucleation rates. And the concentration of hydrogen gas, had also been optimized in our study. We used a 5% hydrogen gas to provide an appropriate reducing atmosphere for the nucleation. The etched sample is annealed again at 600 °C, 700 °C, and 800 °C for 3 h with a rate of 5 °C min^{-1} in H_2 atmosphere (5% H_2 and 95% Ar), which were labeled as sample Bi-TiO_2-600, Bi-TiO_2-700, and Bi-TiO_2-800, respectively.

3. Materials characterization

The powder X-ray diffraction (XRD) patterns were recorded on a Bruker powder diffractometer (Germany) using a Cu-Kα source emitting at 0.15418 nm. Scanning

electron microscopy (SEM) (Phenom Pro Desktop, USA) was used to obtain SEM images. The transmission electron microscope (TEM) and high-resolution TEM (HRTEM) were used on a JEM-2000EX transmission electron microscope with an acceleration voltage of 200 kV. X-ray photoelectron spectroscopy (XPS) was obtained with a Thermo ESCALAB-250 instrument (USA). ^{1}H NMR spectra were recorded on Bruker AVANCE III HD 500 NMR spectrometer. Raman spectra were acquired on a micro-Raman spectrometer using a 532 nm laser as excitation source. The electron paramagnetic resonance (EPR) spectra were recorded using EPR spectrometer (Bruker, Germany).

4. CO_2RR measurement

In electrochemical experiments, the electrochemical workstation (CHI 760E) and a cation exchange membrane (Nafion 117, Dupont) were used. In the H-type cell, 35 mL electrolyte (0.1 M $KHCO_3$), graphite rod, and Ag/AgCl electrode (saturated KCl solution) were used as electrolyte, counter electrode, and reference electrode, respectively. For working electrode, 200 μL of catalyst ink (5 mg mL^{-1}, containing 0.5 wt% Nafion solution) was loaded onto a 1 × 1 cm^2 carbon paper. The electrolyte was degassed by bubbling high-pure CO_2 before the electrochemical tests at least 0.5 h under magnetically stirring (350 rpm) and kept CO_2-saturated during the whole test process at 20 mL min^{-1} by using a mass flow controller. For flow type cell testing, 1 mg cm^{-2} catalyst was loaded gas diffusion electrode as the cathode and Pt sheet was using as the anode for water oxidation. 1 M KOH was using as electrolyte and circulated around the anode and cathode at a flow rate of 10 ml min^{-1} during the CO_2RR. Linear scan voltammetry (LSV) measurements were conducted at −0.2 V to −1.4 V vs. RHE in H-type cell containing 0.1 M $KHCO_3$ or at 0 V to −1.4 V vs. RHE in a gas-diffusion flow cell containing 1 M KOH with a scan rate of 10 mV s^{-1}. Applied potentials were

converted to the reversible hydrogen electrode (RHE) using equation E (vs. RHE) = E (vs. Ag/AgCl) + 0.197 V + 0.059 V × pH. Electrochemical impedance spectra (EIS) were tested in the range of $10^5 \sim 10^{-2}$ Hz with amplitude of 10 mV. The gas products were analyzed by online gas chromatography (GC) systems every 14 min equipped with a thermal conductivity detector and flame ionization detectors, and high purity argon gas (99.999%) was used as the carrier gas for the GC. The liquid products were analyzed using NMR spectroscopy by adding 630 μL electrolyte into the 70 μL D_2O (deuterated water) containing dimethyl sulfoxide solution (DMSO, as an internal standard) with water peak suppression method.

Faradaic efficiency: The Faradaic efficiency (FE) can be calculated as the following equation: FE = wnF / Q

where, *w* is the number of electrons transfer involved in the reaction (for example, two-electron transfer for formate, CO, and H_2 products), *n* is the number of moles of product formed over time, F is the Faraday constant, and Q is the total amount of charges through the CO_2RR process in this time.

5. In situ ATR-FTIR

In situ ATR-FTIR experiments were performed using a HgCdTe (MCT) detector equipped with liquid nitrogen cooling and purification with dry air. A PIKE electrochemical cell was mounted on a VeeMax III ATR accessory with 60° Au-coated silicon prism loaded catalysts. All ATR-FTIR measurements had a spectral resolution of 4 cm^{-1}, an optical velocity of 1.8988, and a gain of 1. Three-electrode electrochemical system with the silicon prisms as work electrode, Ag/AgCl as reference electrode, and Pt wires counter electrodes was used.

6. DFT calculations

Density functional theory (DFT) calculations are carried out in this work based on the CASTEP packages to investigate the size effects of Bi nanoparticles on TiO_2.[1] For all the exchange-correlation interactions, the generalized gradient approximation (GGA) and Perdew-Burke-Ernzerhof (PBE) functionals are chosen to realize the sufficient description.[2-4] Meanwhile, a 380 eV cutoff energy has been applied for the calculations, which is generated based on the ultrafine quality of cutoff energy and ultrasoft pseudopotentials. In addition, we adopt the Broyden-Fletcher-Goldfarb-Shannon (BFGS) algorithm with coarse *k*-point settings for all the energy minimization.[5] The convergence tests have proved that these settings are sufficient to reveal the changes in both energy and electronic structures. Considering the computational loadings and efficiency, we have cleaved the Bi nanoparticles from the (104) surface with 10, 14, and 26 atoms of Bi atoms in Bi-TiO_2-800, Bi-TiO_2-700, and Bi-TiO_2-600, respectively. The nanoparticle sizes are 0.59 nm, 0.80 nm, and 1.19 nm, respectively, which are considered as small, medium, and large sizes to investigate the size effect. TiO_2 is cleaved from (100) surfaces of anatase TiO_2 with four atomic layers with 144 atoms. Moreover, the 20 Å vacuum space is introduced in the *z*-axis direction to supply sufficient space on the catalyst surface for all the relaxations. Moreover, we have applied strict convergence requirements for all the calculations in this work as follows. The Hellmann-Feynman forces and total energy difference should not exceed 1×10^{-3} eV/Å and 5×10^{-5} eV/atom, respectively.

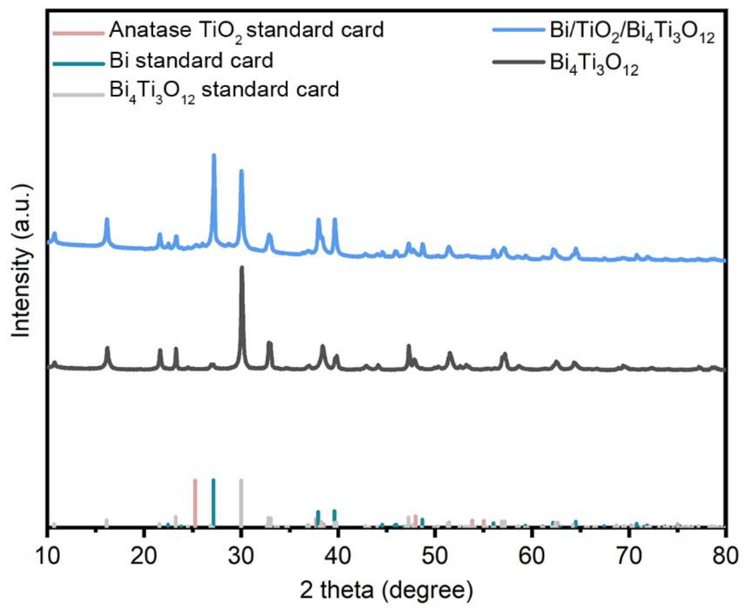

Figure S1. XRD patterns of first annealed $Bi/TiO_2/Bi_4Ti_3O_{12}$, $Bi_4Ti_3O_{12}$, and the standard PDF cards of anatase TiO_2, Bi metal, and $Bi_4Ti_3O_{12}$.

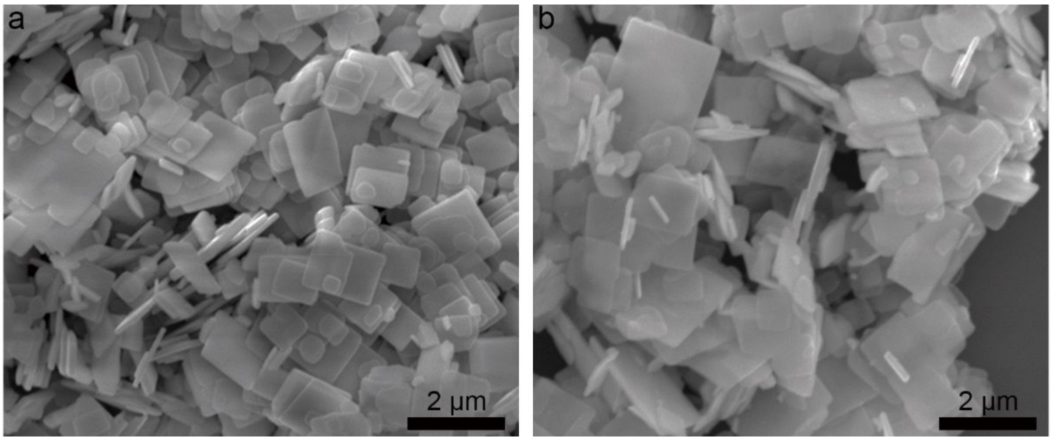

Figure S2. The SEM images of (a) $Bi_4Ti_3O_{12}$ and (b) $Bi/TiO_2/Bi_4Ti_3O_{12}$.

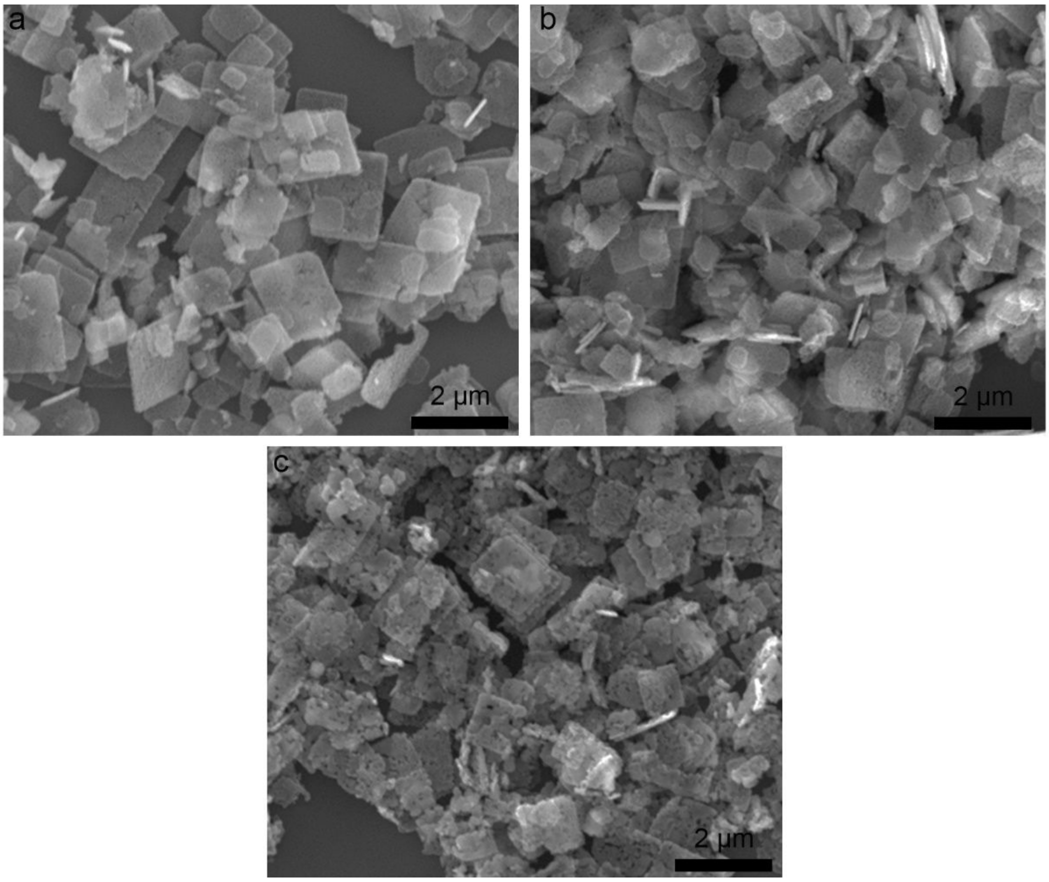

Figure S3. The SEM images of (a) Bi-TiO_2-600, (b) Bi-TiO_2-700, and (c) Bi-TiO_2-800.

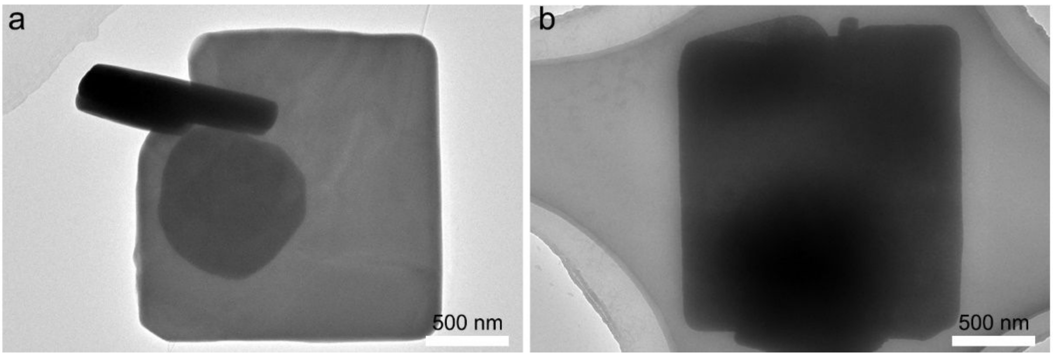

Figure S4. The TEM images of (a) $Bi_4Ti_3O_{12}$ and (b) $Bi/TiO_2/Bi_4Ti_3O_{12}$.

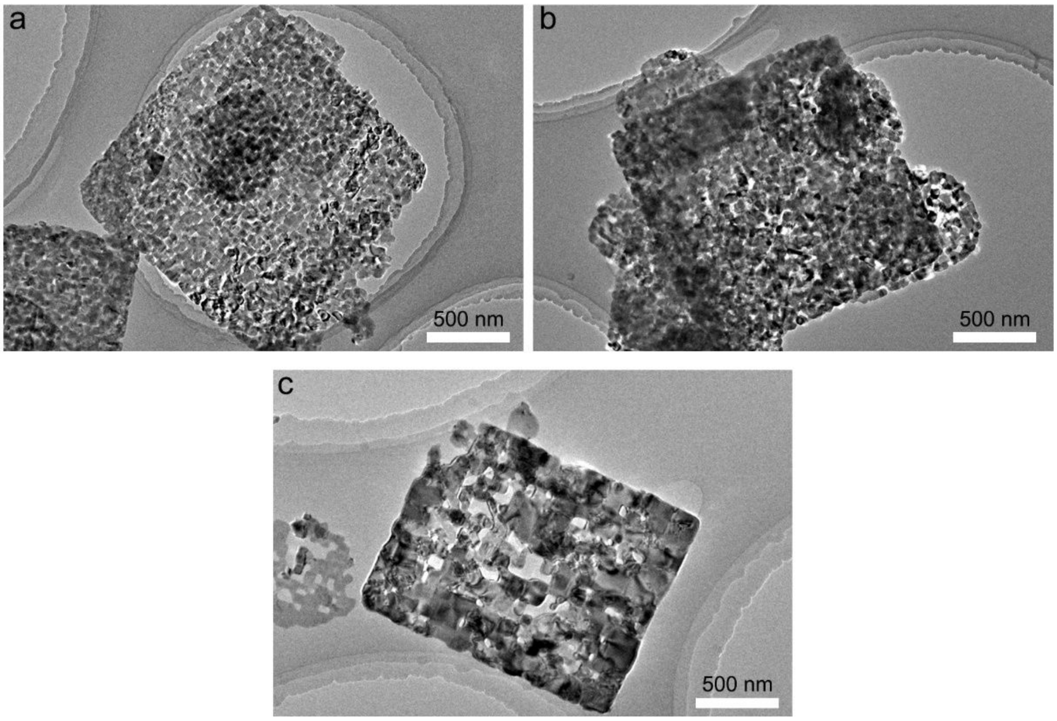

Figure S5. The TEM images of (a) Bi-TiO_2-600, (b) Bi-TiO_2-700, and (c) Bi-TiO_2-800.

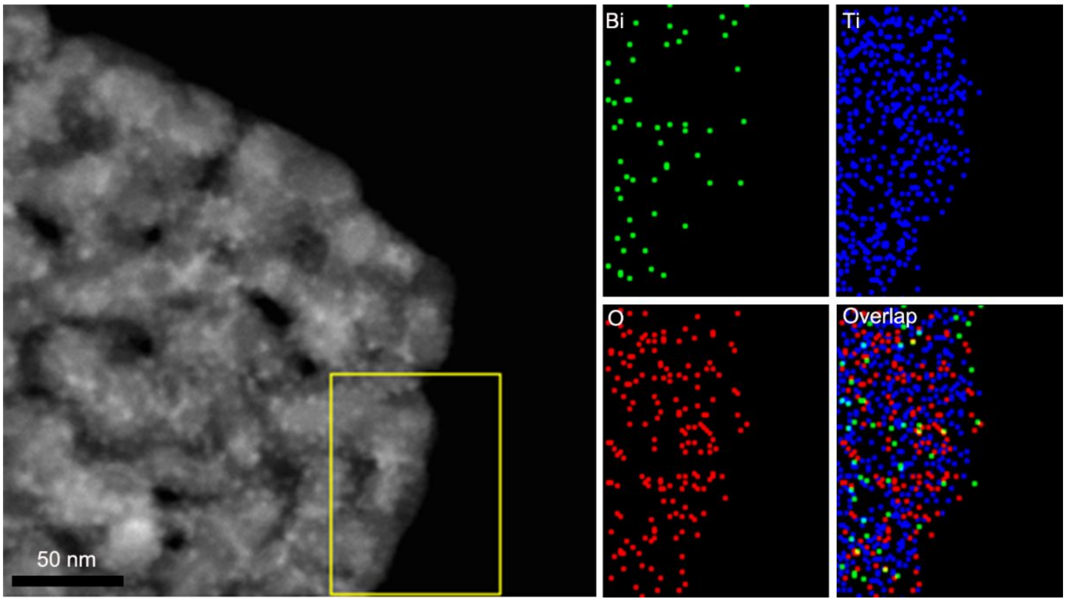

Figure S6. The high-angle annular dark-field transmission electron microscopy (HAADF-TEM) images and corresponding element mapping of Bi-TiO_2-700.

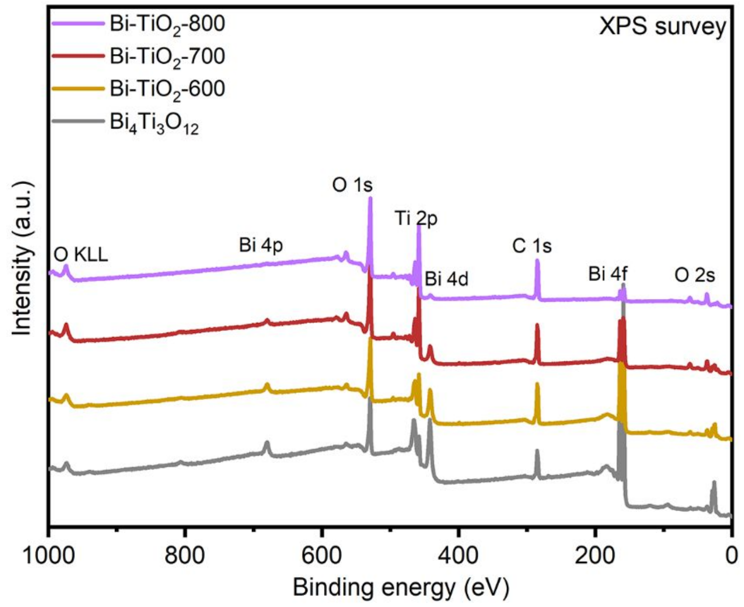

Figure S7. XPS survey of $Bi_4Ti_3O_{12}$, Bi-TiO_2-600, Bi-TiO_2-700, and Bi-TiO_2-800.

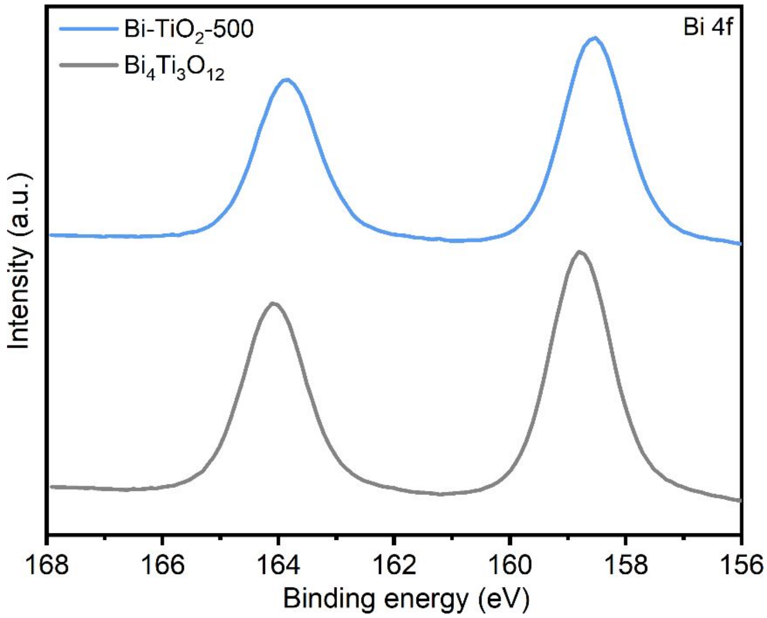

Figure S8. XPS spectra of Bi 4f of $Bi_4Ti_3O_{12}$ and $Bi/TiO_2/Bi_4Ti_3O_{12}$.

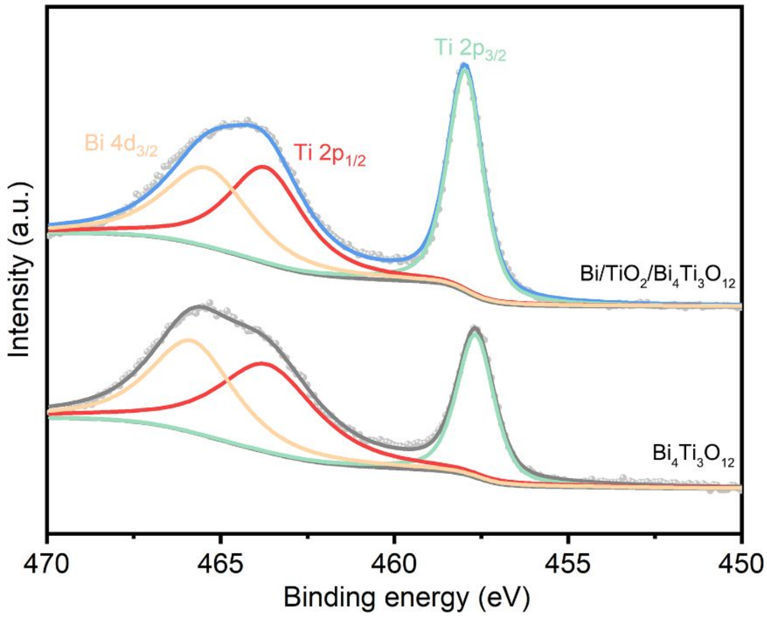

Figure S9. XPS spectra of Ti 2p of $Bi_4Ti_3O_{12}$ and $Bi/TiO_2/Bi_4Ti_3O_{12}$.

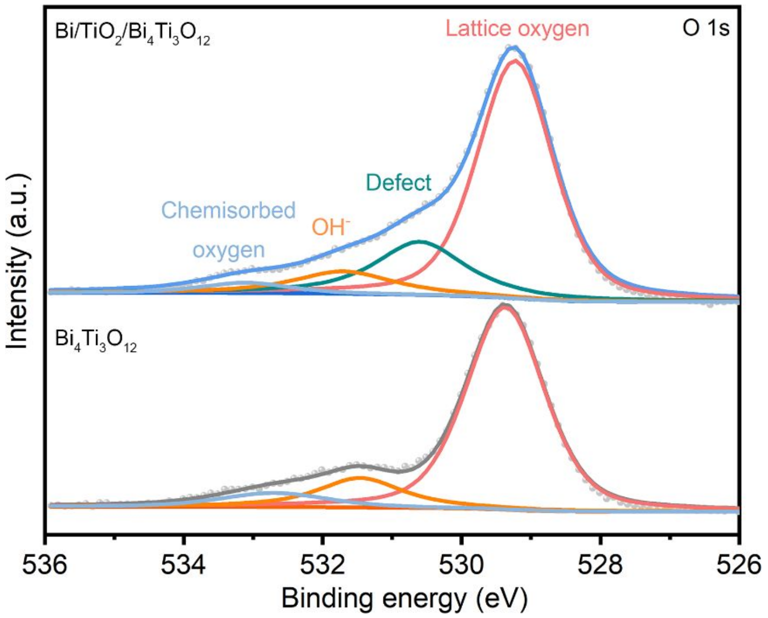

Figure S10. XPS spectra of O1s of $Bi_4Ti_3O_{12}$ and $Bi/TiO_2/Bi_4Ti_3O_{12}$.

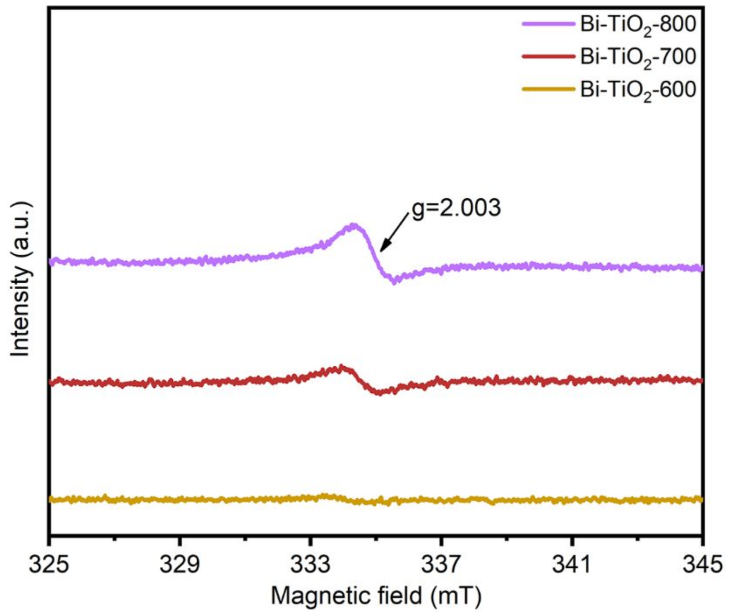

Figure S11. EPR spectra of Bi-TiO_2-600, Bi-TiO_2-700, and Bi-TiO_2-800.

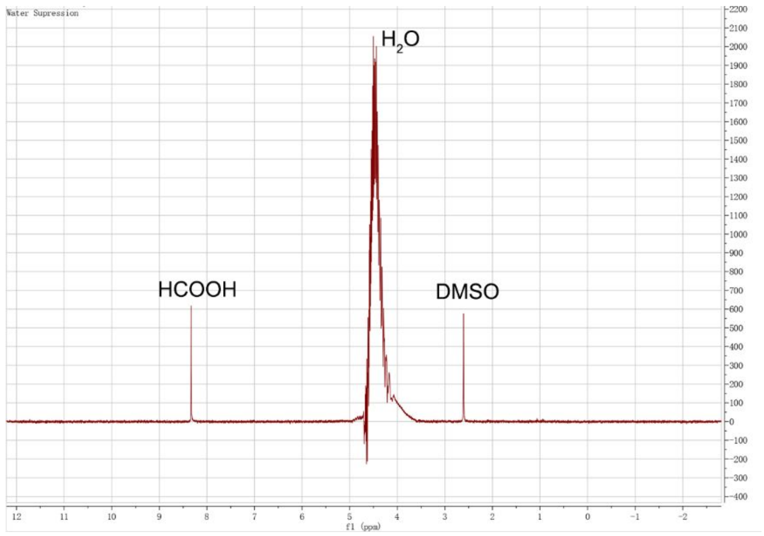

Figure S12. ^{1}H NMR spectrum of liquid product.

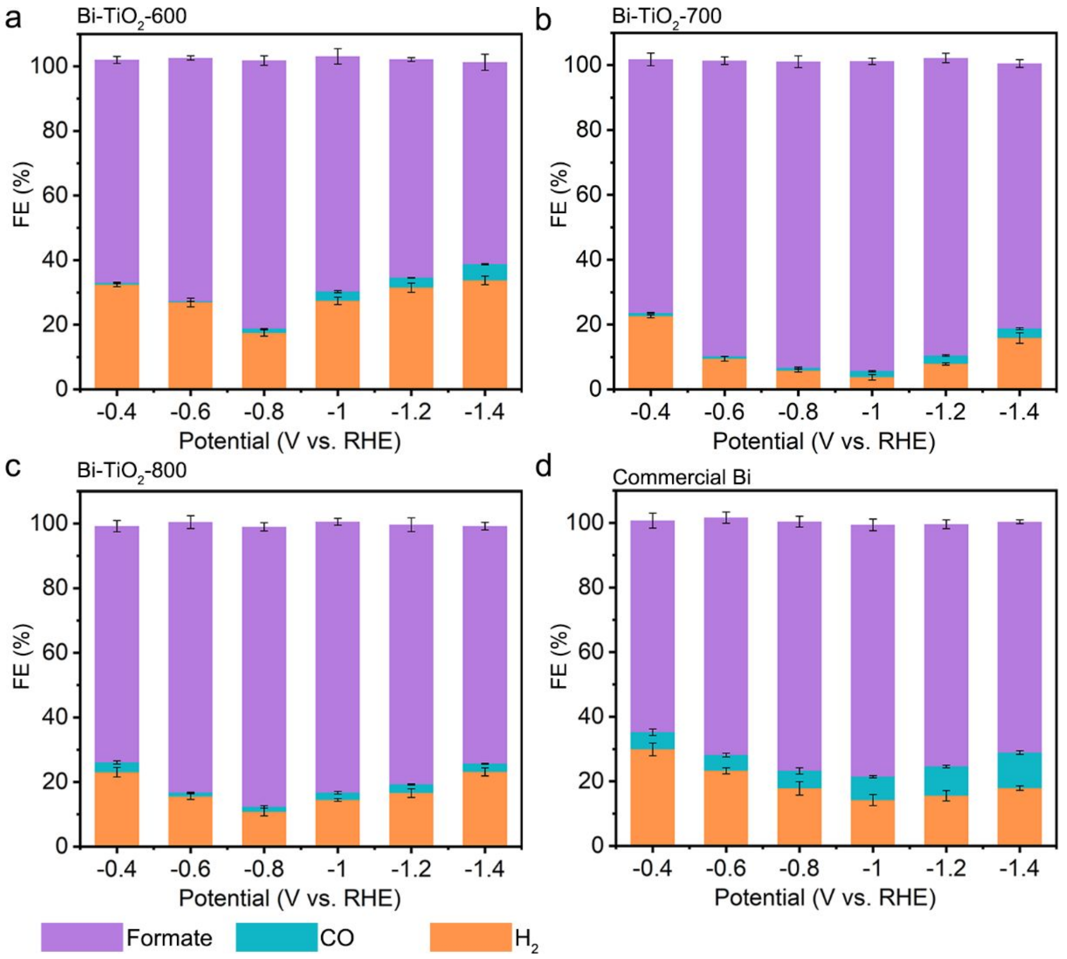

Figure S13. FEs of products on (a) Bi-TiO_2-600, (b) Bi-TiO_2-700, (c) Bi-TiO_2-800, and (d) commercial Bi catalysts at different potentials for CO_2RR.

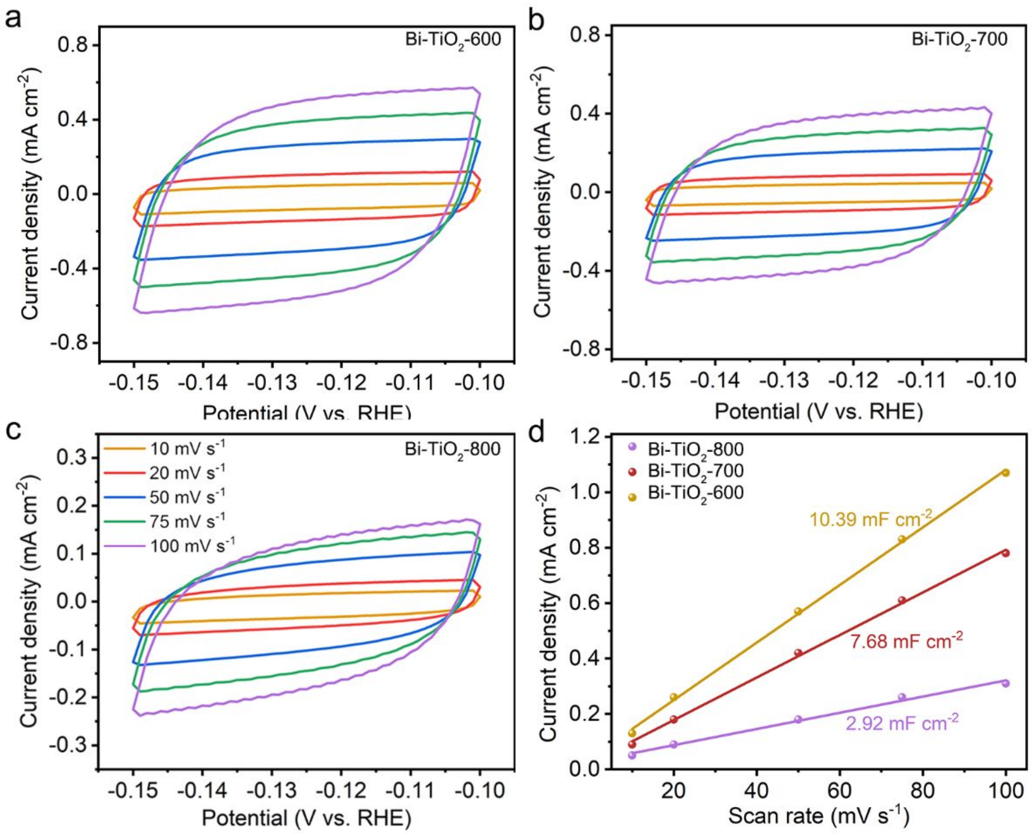

Figure S14. Effective electrochemical active surface area (ECSA) tests: cyclic voltammogram curves of (a) Bi-TiO_2-600, (b) Bi-TiO_2-700, and (c) Bi-TiO_2-800, and (d) capacitance values.

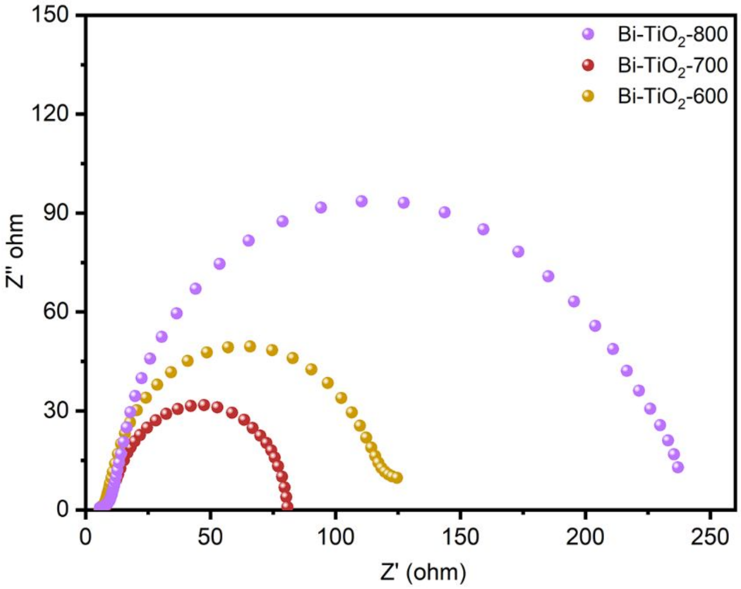

Figure S15. EIS of Bi-TiO_2-600, Bi-TiO_2-700, and Bi-TiO_2-800.

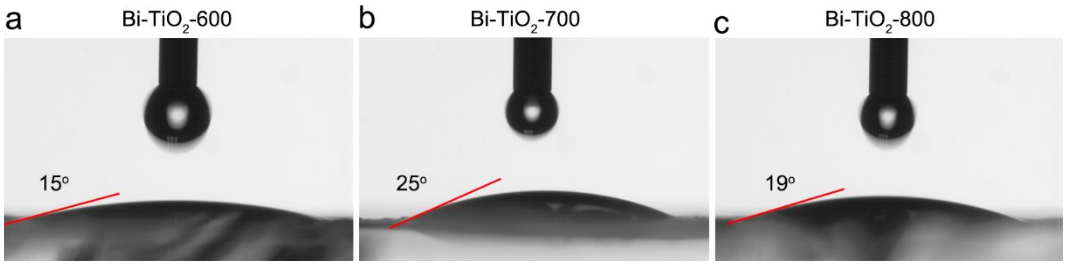

Figure S16. Characterization of the hydrophilicity of the (a) Bi-TiO_2-600, (b) Bi-TiO_2-700, and (c) Bi-TiO_2-800 catalysts using water contact angles by the tangent method.

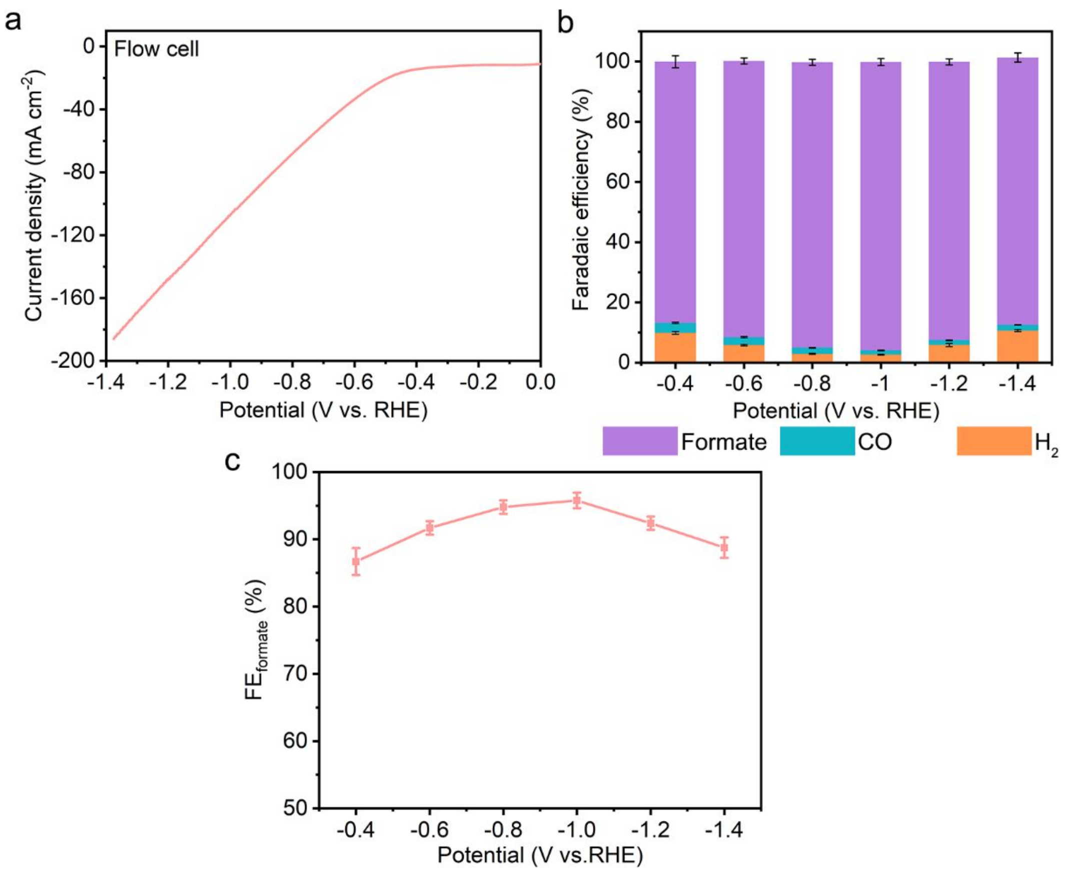

Figure S17. (a) LSV and (b) FEs of products on Bi-TiO_2-700 in 1 M KOH using a gas-diffusion flow cell. (c) FEs of formate of Bi-TiO_2-700 in 1 M KOH using a gas-diffusion flow cell.

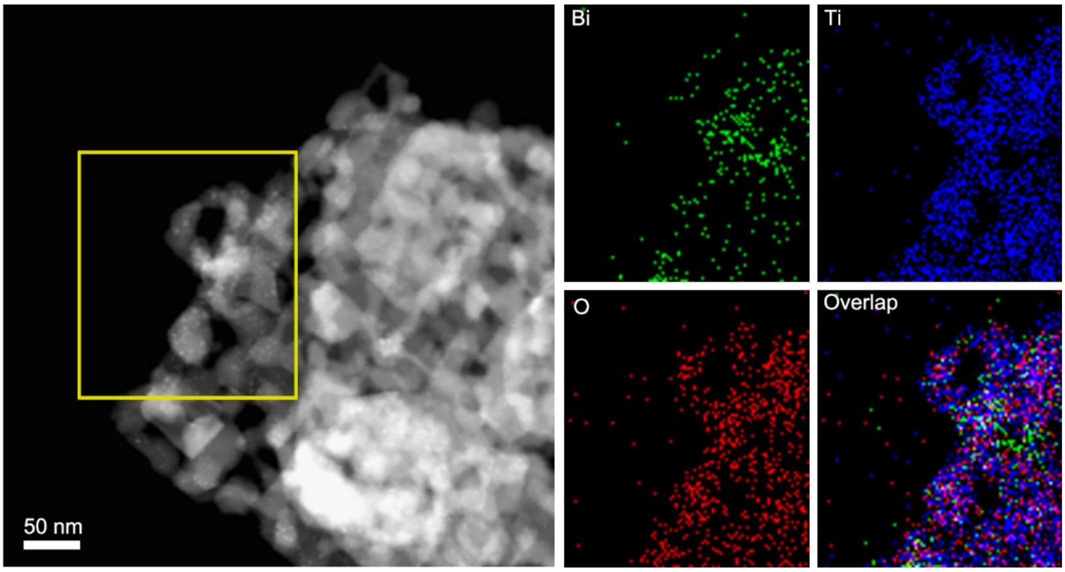

Figure S18. The HAADF-TEM and corresponding element mapping of Bi-TiO_2-700 after electrochemical CO_2RR.

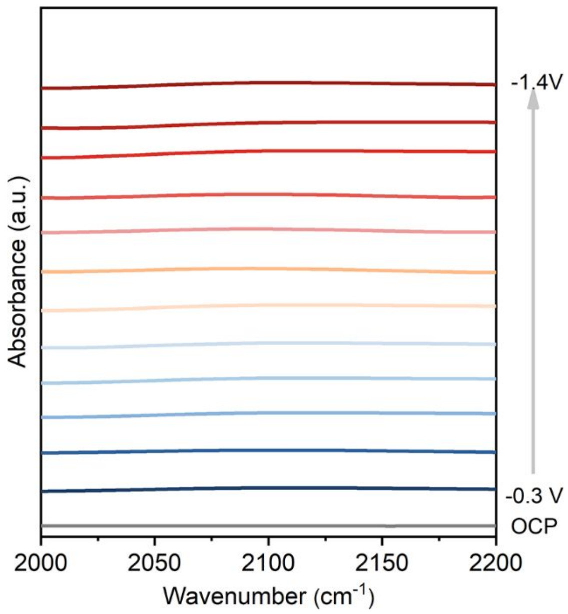

Figure S19. In situ ATR-FTIR spectra collected of Bi-TiO_2-700 at different applied potentials from −0.3 V to −1.4 V vs RHE.

Table S1. The content of Bi of Bi-TiO_2-600, Bi-TiO_2-700, and Bi-TiO_2-800.

Samples	Bi-TiO_2-600	Bi-TiO_2-700	Bi-TiO_2-800
**Bi content (at%)**	2.89	2.84	2.86
**Ti content (at%)**	32.65	32.52	32.45

Table S2. Summary of the performances of Bi-based electrocatalysts for CO_2RR in H-type.

Materials	Electrolytes	Potentials (V vs. RHE)	FE_{HCOOH} (%)	$-J_{HCOOH}$ (mA/cm^2)	Ref.
**Bi-TiO_2**	0.1 M $KHCO_3$	–1.0	95.6	6.7	This work
		–1.2	91.8	12.3	
**Bi_2O_3@C**	0.5 M $KHCO_3$	–0.9	93	7.5	S1[6]
**Bi-Sn aerogel**	0.1 M $KHCO_3$	–1.0	93.9	9.3	S2[7]
**Bi@C**	0.5 M $KHCO_3$	–0.99	95.0	10.5	S3[8]
**Bismuth subcarbonate**	0.5 M $KHCO_3$	–0.7	85	9.3	S4[9]
**Bi nanosheet**	0.1 M $KHCO_3$	–0.8	97	~3	S5[10]
**$Ag_{0.95}BiS_{0.75}O_{3.1}$**	0.1 M $KHCO_3$	–0.7	94.3	12.52	S6[11]
**Bi-based MOF**	0.5 M $KHCO_3$	–0.97	95	5.4	S7[12]
**Bi@C nanotubes**	0.1 M $KHCO_3$	–0.9	90.9	5.3	S8[13]
**Ultrathin Bi nanosheet array**	0.1 M $KHCO_3$	–1.1	86.0	16.5	S9[14]
**Bi-MOFs**	0.1 M $KHCO_3$	–1.1	92	10.5	S10[15]
**Bi-Sn nano-alloys**	0.1 M $KHCO_3$	–1.1	78	10.7	S11[16]
**BiO_x-SnO_x nanoflakes**	0.5 M $KHCO_3$	–1.37	90.8	40.9	S12[17]
**Bi_2O_3**	0.5 M $KHCO_3$	–0.9	91	8	S13[18]
**Bi dendrite**	0.5 M $KHCO_3$	–0.74	89	2.7	S14[19]
**Bi_2Te_3 nanoplates**	0.5 M $KHCO_3$	–0.9	89.6	~13	S15[20]
**Bi_2S_3–Bi_2O_3@rGO**	0.1 M $KHCO_3$	–0.9	90.1	~35.0	S16[21]
**Bi nanosheet**	0.5 M $KHCO_3$	–0.79	99.0	17.3	S17[22]
**Bi@Sn core-shell**	0.5 M $KHCO_3$	–1.1	91	32	S18[23]
**Atomically thin Bismuthene**	0.5 M $KHCO_3$	–0.83	97.4	15.3	S19[24]
**Bi nanowires on Cu foam**	0.5 M $KHCO_3$	–0.99	95	45	S20[25]
**Bi/Bi_2O_3 nanosheets**	0.5 M $KHCO_3$	−0.87	90.4	32.4	S21[26]
**Bismuthene**	0.5 M $KHCO_3$	−0.9	95	100	S22[27]

Table S3. Summary of the performances of Bi-based electrocatalysts for CO_2RR in flow cell.

Materials	Electrolytes	Potentials (V vs. RHE)	FE_{HCOOH} (%)	$-J_{HCOOH}$ (mA/cm^2)	Ref.
**Bi-TiO_2**	1 M KOH	−1.0	95.8	102.6	This work
**Sn-Bi/SnO_2**	1 M KOH	−0.62	95	95	[28]
**Bismuthene arrays**	1 M $KHCO_3$	−1.2	93.8	~200	[29]
**Bi rhombic dodecahedra**	1 M KOH	−0.68	86	~200	[30]
**Bi nanosheets**	0.5 M $KHCO_3$	−1.4	95.2	72	[31]
**Bi-doped amorphous SnO_x**	0.5 M $KHCO_3$	−0.88	95.8	20.9	[32]

References

(1) Clark, S. J.; Segall, M. D.; Pickard, C. J.; Hasnip, P. J.; Probert, M. I. J.; Refson, K.; Payne, M. C., First Principles Methods Using Castep. *Z. Kristallogr.* **2005,** *220* (5-6), 567-570.

(2) Perdew, J. P.; Burke, K.; Ernzerhof, M., Generalized Gradient Approximation Made Simple. *Phys. Rev. Lett.* **1996,** *77* (18), 3865-3868.

(3) Hasnip, P. J.; Pickard, C. J., Electronic Energy Minimisation with Ultrasoft Pseudopotentials. *Comput. Phys. Commun.* **2006,** *174* (1), 24-29.

(4) Perdew, J. P.; Chevary, J. A.; Vosko, S. H.; Jackson, K. A.; Pederson, M. R.; Singh, D. J.; Fiolhais, C., Atoms, Molecules, Solids, and Surfaces: Applications of the Generalized Gradient Approximation for Exchange and Correlation. *Phys. Rev. B* **1992,** *46* (11), 6671-6687.

(5) Head, J. D.; Zerner, M. C., A Broyden-Fletcher-Goldfarb-Shanno Optimization Procedure for Molecular Geometries. *Chem. Phys. Lett.* **1985,** *122* (3), 264-270.

(6) Deng, P.; Yang, F.; Wang, Z.; Chen, S.; Zhou, Y.; Zaman, S.; Xia, B. Y., Metal-Organic Framework-Derived Carbon Nanorods Encapsulating Bismuth Oxides for Rapid and Selective CO_2 Electroreduction to Formate. *Angew. Chem., Int. Ed.* **2020,** *59* (27), 10807-10813.

(7) Wu, Z.; Wu, H.; Cai, W.; Wen, Z.; Jia, B.; Wang, L.; Jin, W.; Ma, T., Engineering Bismuth–Tin Interface in Bimetallic Aerogel with a 3d Porous Structure for Highly Selective Electrocatalytic CO_2 Reduction to Hcooh. *Angew. Chem., Int. Ed.* **2021,** *60* (22), 12554-12559.

(8) Liu, S.; Fan, Y.; Wang, Y.; Jin, S.; Hou, M.; Zeng, W.; Li, K.; Jiang, T.; Qin, L.; Yan, Z.; Tao, Z.; Zheng, X.; Shen, C.; Liu, Z.; Ahmad, T.; Zhang, K.; Chen, W., Surface-Oxygen-Rich Bi@C Nanoparticles for High-Efficiency Electroreduction of CO_2 to Formate. *Nano Lett.* **2022,** *22* (22), 9107-9114.

(9) Zhang, Y.; Zhang, X.; Ling, Y.; Li, F.; Bond, A. M.; Zhang, J., Controllable Synthesis of Few-Layer Bismuth Subcarbonate by Electrochemical Exfoliation for Enhanced CO_2 Reduction Performance. *Angew. Chem., Int. Ed.* **2018,** *57* (40), 13283-13287.

(10) Lu, P.; Gao, D.; He, H.; Wang, Q.; Liu, Z.; Dipazir, S.; Yuan, M.; Zu, W.; Zhang, G., Facile Synthesis of a Bismuth Nanostructure with Enhanced Selectivity for Electrochemical Conversion of CO_2 to Formate. *Nanoscale* **2019,** *11* (16), 7805-7812.

(11) Zhou, J.-H.; Yuan, K.; Zhou, L.; Guo, Y.; Luo, M.-Y.; Guo, X.-Y.; Meng, Q.-Y.; Zhang, Y.-W., Boosting Electrochemical Reduction of CO_2 at a Low Overpotential by Amorphous Ag-Bi-S-O Decorated Bi^0 Nanocrystals. *Angew. Chem., Int. Ed.* **2019,** *58* (40), 14197-14201.

(12) Lamagni, P.; Miola, M.; Catalano, J.; Hvid, M. S.; Mamakhel, M. A. H.; Christensen, M.; Madsen, M. R.; Jeppesen, H. S.; Hu, X.-M.; Daasbjerg, K.; Skrydstrup, T.; Lock, N., Restructuring Metal-Organic Frameworks to Nanoscale Bismuth Electrocatalysts for Highly Active and Selective CO_2 Reduction to Formate. *Adv. Funct. Mater.* **2020,** *30* (16), 1910408.

(13) Zhang, W.; Yang, S.; Jiang, M.; Hu, Y.; Hu, C.; Zhang, X.; Jin, Z., Nanocapillarity and Nanoconfinement Effects of Pipet-Like Bismuth@Carbon Nanotubes for Highly Efficient Electrocatalytic CO_2 Reduction. *Nano Lett.* **2021,** *21* (6), 2650-2657.

(14) Zhang, W.; Hu, Y.; Ma, L.; Zhu, G.; Zhao, P.; Xue, X.; Chen, R.; Yang, S.; Ma, J.; Liu, J.; Jin, Z., Liquid-Phase Exfoliated Ultrathin Bi Nanosheets: Uncovering the Origins of Enhanced Electrocatalytic CO_2 Reduction on Two-Dimensional Metal Nanostructure. *Nano Energy* **2018,** *53*, 808-816.

(15) Yao, D.; Tang, C.; Vasileff, A.; Zhi, X.; Jiao, Y.; Qiao, S.-Z., The Controllable Reconstruction of Bi-Mofs for Electrochemical CO_2 Reduction through Electrolyte and Potential Mediation. *Angew.*

Chem., Int. Ed. **2021,** *60* (33), 18178-18184.

(16) Tang, J.; Daiyan, R.; Ghasemian, M. B.; Idrus-Saidi, S. A.; Zavabeti, A.; Daeneke, T.; Yang, J.; Koshy, P.; Cheong, S.; Tilley, R. D.; Kaner, R. B.; Amal, R.; Kalantar-Zadeh, K., Advantages of Eutectic Alloys for Creating Catalysts in the Realm of Nanotechnology-Enabled Metallurgy. *Nat. Commun.* **2019,** *10* (1), 4645.

(17) Yuan, T.; Hu, Z.; Zhao, Y.; Fang, J.; Lv, J.; Zhang, Q.; Zhuang, Z.; Gu, L.; Hu, S., Two-Dimensional Amorphous Snox from Liquid Metal: Mass Production, Phase Transfer, and Electrocatalytic CO_2 Reduction toward Formic Acid. *Nano Lett.* **2020,** *20* (4), 2916-2922.

(18) Deng, P.; Wang, H.; Qi, R.; Zhu, J.; Chen, S.; Yang, F.; Zhou, L.; Qi, K.; Liu, H.; Xia, B. Y., Bismuth Oxides with Enhanced Bismuth-Oxygen Structure for Efficient Electrochemical Reduction of Carbon Dioxide to Formate. *ACS Catal.* **2020,** *10* (1), 743-750.

(19) Koh, J. H.; Won, D. H.; Eom, T.; Kim, N.-K.; Jung, K. D.; Kim, H.; Hwang, Y. J.; Min, B. K., Facile CO_2 Electro-Reduction to Formate Via Oxygen Bidentate Intermediate Stabilized by High-Index Planes of Bi Dendrite Catalyst. *ACS Catal.* **2017,** *7* (8), 5071-5077.

(20) Zhang, N.; Zheng, F.; Huang, B.; Ji, Y.; Shao, Q.; Li, Y.; Xiao, X.; Huang, X., Exploring Bi_2Te_3 Nanoplates as Versatile Catalysts for Electrochemical Reduction of Small Molecules. *Adv. Mater.* **2020,** *32* (22), 1906477.

(21) Yang, X.; Deng, P.; Liu, D.; Zhao, S.; Li, D.; Wu, H.; Ma, Y.; Xia, B. Y.; Li, M.; Xiao, C.; Ding, S., Partial Sulfuration-Induced Defect and Interface Tailoring on Bismuth Oxide for Promoting Electrocatalytic CO_2 Reduction. *J. Mater. Chem. A* **2020,** *8* (5), 2472-2480.

(22) Xia, C.; Zhu, P.; Jiang, Q.; Pan, Y.; Liang, W.; Stavitski, E.; Alshareef, H. N.; Wang, H., Continuous Production of Pure Liquid Fuel Solutions Via Electrocatalytic CO_2 Reduction Using Solid-Electrolyte Devices. *Nat. Energy* **2019,** *4* (9), 776-785.

(23) Xing, Y.; Kong, X.; Guo, X.; Liu, Y.; Li, Q.; Zhang, Y.; Sheng, Y.; Yang, X.; Geng, Z.; Zeng, J., Bi@Sn Core-Shell Structure with Compressive Strain Boosts the Electroreduction of CO_2 into Formic Acid. *Adv. Sci.* **2020,** *7* (22), 1902989.

(24) Cao, C.; Ma, D.-D.; Gu, J.-F.; Xie, X.; Zeng, G.; Li, X.; Han, S.-G.; Zhu, Q.-L.; Wu, X.-T.; Xu, Q., Metal–Organic Layers Leading to Atomically Thin Bismuthene for Efficient Carbon Dioxide Electroreduction to Liquid Fuel. *Angew. Chem., Int. Ed.* **2020,** *59* (35), 15014-15020.

(25) Zhang, X.; Sun, X.; Guo, S.-X.; Bond, A. M.; Zhang, J., Formation of Lattice-Dislocated Bismuth Nanowires on Copper Foam for Enhanced Electrocatalytic CO_2 Reduction at Low Overpotential. *Energy Environ. Sci.* **2019,** *12* (4), 1334-1340.

(26) Wu, D.; Huo, G.; Chen, W.; Fu, X.-Z.; Luo, J.-L., Boosting Formate Production at High Current Density from CO_2 Electroreduction on Defect-Rich Hierarchical Mesoporous Bi/Bi_2O_3 Junction Nanosheets. *Appl. Catal., B* **2020,** *271*, 118957.

(27) Su, L.; Wang, P.; Wang, J.; Zhang, D.; Wang, H.; Li, Y.; Zhan, S.; Gong, J., Pt-Cu Interaction Induced Construction of Single Pt Sites for Synchronous Electron Capture and Transfer in Photocatalysis. *Adv. Funct. Mater.* **2021,** *31* (47), 2104343.

(28) Li, L.; Ozden, A.; Guo, S.; García de Arquer, F. P.; Wang, C.; Zhang, M.; Zhang, J.; Jiang, H.; Wang, W.; Dong, H.; Sinton, D.; Sargent, E. H.; Zhong, M., Stable, Active CO_2 Reduction to Formate Via Redox-Modulated Stabilization of Active Sites. *Nat. Commun.* **2021,** *12* (1), 5223.

(29) He, Y.-C.; Ma, D.-D.; Zhou, S.-H.; Zhang, M.; Tian, J.-J.; Zhu, Q.-L., Integrated 3d Open Network of Interconnected Bismuthene Arrays for Energy-Efficient and Electrosynthesis-Assisted Electrocatalytic CO_2 Reduction. *Small* **2022,** *18* (1), 2105246.

(30) Xie, H.; Zhang, T.; Xie, R.; Hou, Z.; Ji, X.; Pang, Y.; Chen, S.; Titirici, M.-M.; Weng, H.; Chai, G., Facet Engineering to Regulate Surface States of Topological Crystalline Insulator Bismuth Rhombic Dodecahedrons for Highly Energy Efficient Electrochemical CO_2 Reduction. *Adv. Mater.* **2021,** *33* (31), 2008373.

(31) Fu, X.; Wang, J.-a.; Hu, X.; He, K.; Tu, Q.; Yue, Q.; Kang, Y., Scalable Chemical Interface Confinement Reduction Biobr to Bismuth Porous Nanosheets for Electroreduction of Carbon Dioxide to Liquid Fuel. *Adv. Funct. Mater.* **2022,** *32* (10), 2107182.

(32) Yang, Q.; Wu, Q.; Liu, Y.; Luo, S.; Wu, X.; Zhao, X.; Zou, H.; Long, B.; Chen, W.; Liao, Y.; Li, L.; Shen, P. K.; Duan, L.; Quan, Z., Novel Bi-Doped Amorphous Snox Nanoshells for Efficient Electrochemical CO_2 Reduction into Formate at Low Overpotentials. *Adv. Mater.* **2020,** *32* (36), 2002822.
